# Regulation of TGF-β1-induced fibroblast differentiation of human periodontal ligament stem cells through the mutually antagonistic action of ectonucleotide pyrophosphatase/phosphodiesterase 1 and 2

**DOI:** 10.3389/fcell.2024.1426762

**Published:** 2024-09-03

**Authors:** Onyou Ju, Seon-Yle Ko, Young-Joo Jang

**Affiliations:** ^1^ Department of Nanobiomedical Science and BK21 FOUR NBM Global Research Center for Regenerative Medicine, Dankook University, Cheonan, Republic of Korea; ^2^ Department of Oral Biochemistry, School of Dentistry, Dankook University, Cheonan, Republic of Korea

**Keywords:** periodontal ligament stem cells (PDLSC), TGF-β1, fibroblastic differentiation, ENPP1, ENPP2

## Abstract

Human periodontal ligament stem cells (hPDLSCs) differentiate into periodontal ligament (PDL) fibroblasts, osteoblasts, and cementoblasts. To identify inducers of PDL fibroblastic differentiation, monoclonal antibody series were developed a series of against membrane/extracellular matrix (ECM) molecules through decoy immunization. The anti-PDL13 antibody targets ectonucleotide pyrophosphatase/phosphodiesterase 1 (ENPP1), renowned for regulating skeletal and soft tissue mineralization. ENPP1 accumulates in the periodontal ligament region of tooth roots, and specifically localizes to the cell boundaries and elongated processes of the fibroblastic cells. As ENPP1 expression increases during fibroblastic differentiation, mineralization induced by tissue-nonspecific alkaline phosphatase (TNAP), a pyrophosphate-degrading enzyme, is completely inhibited. This is consistent with ENPP1 and TNAP acting in opposition, and TGF-β1-induced ENPP1 expression creates an essential environment for PDL fibroblast differentiation. Representative fibroblastic differentiation markers decrease with endogenous ENPP1 inhibition by siRNA and antibody blocking. ENPP2 generates lipid signaling molecules. In contrast to ENPP1, ENPP2 disappears in TGF-β1-induced PDL fibroblasts. Ectopic expression of *ENPP2* hinders TGF-β1-induced PDL fibroblastic differentiation. Suppression of ENPP1 and ENPP2 leads to severe defects in undifferentiated and differentiated cells, demonstrating that these two factors play opposing roles in soft and hard tissue differentiation but can complement each other for cell survival. In conclusion, increased ENPP1 is crucial for TGF-β1-induced PDL differentiation, while ENPP2 and TNAP can inhibit ENPP1. ENPP1 and ENPP2 exhibit complementary functions in the cell survival.

## Introduction

The teeth are supported at the root by the periodontal tissue, comprising the cementum, periodontal ligament (PDL), and alveolar bone. The PDL, a connective tissue located between two mineralized tissues, the alveolar bone and cementum. It plays a crucial role in anchoring tooth roots into the bone socket. Postnatal stem cells that differentiate into osteoblasts, cementoblasts, and PDL fibroblasts have been identified within PDL tissues (Seo et al., 2004; Chen et al., 2006). Given the PDL’s location between two hard tissues, periodontal ligament stem cells (PDLSCs) must inhibit mineralization and differentiate properly into soft tissues during PDL regeneration. While PDLSCs cultured *in vitro* can express osteogenic and cementogenic markers under physiological conditions, periodontal tissue typically remains in an unmineralized fibrous state. This observation suggests that the fibroblastic state is dominant, and osteo/cementoblastic differentiation is generally suppressed in PDLSCs (Yamada et al., 2007; Choi et al., 2015; Hyun et al., 2017; Lim et al., 2020). TGF-β1 plays a crucial role in promoting the production of fibrogenic collagens and regulating fibroblastic tissue formation during development (Arai et al., 2002). Its expression is higher in the PDL compared to the cementum and alveolar bone, where it inhibits BMP2-induced hard tissue formation (Li et al., 2014; Kawahara et al., 2015). Blocking TGF-β1 signaling while activating the BMP signaling pathway can create optimal conditions for inducing cementogenic differentiation in hPDLSCs (Lim et al., 2020).

However, in periodontal regeneration studies, the width of the newly formed PDL diminishes over time because of mineralization occurring at the interfaces between PDL-cementum and PDL-alveolar bone. This suggests that the instability of the regenerated PDL could eventually rest in mineralization over the long term (Liu et al., 2016). Hence, the precise control of mineralization is crucial for periodontal tissue regeneration. Currently, the mechanism of PDL tissue maintenance through the suppression of mineralization is not clearly known.

Biomineralization is regulated by the ratio of inorganic phosphate (Pi) to pyrophosphate (PPi), defining the properties of inorganic materials. PPi has been studied as a mineralization inhibitor, directly binding to crystals to impede further mineral growth (Addison et al., 2007; Foster et al., 2012). Understanding the role of PPi in PDLSCs is crucial for comprehending PDL maintenance in periodontal tissue, which forms the basis for the long-term stability of regenerative PDL. PPi inhibits osteogenic differentiation *in vitro* by activating ERK1/2, JNK, and p38 signaling pathways. Treatment with a p38 inhibitor increases the expression of osteoblast-related genes, whereas this inhibitor does not recover their expression under PPi treatment (Foster et al., 2012; Liang et al., 2021). Extracellular Pi and PPi concentrations are regulated by phosphatases, including tissue-nonspecific alkaline phosphatase (TNAP) and ectonucleotide pyrophosphatase/phosphodiesterase 1 (ENPP1) (Hessle et al., 2002; McKee et al., 2013; Millan, 2013). Loss of *TNAP* inhibits cementum formation, whereas loss of *ENPP1* promotes acellular cementum formation. These findings indicate that early TNAP expression creates a low-PPi environment conductive to acellular cementum initiation, while ENPP1 expression increases PPi levels, thereby limiting, acellular cementum hyperplasia (Zweifler et al., 2015).

The ENPP family consists of seven members (ENPP1-7) and their roles in regulating extracellular PPi levels, cell migration, and angiogenesis have been extensively researched (Goding et al., 2003; Stefan et al., 2005; Borza et al., 2022). Structural differences in the substrate-binding site determine that ENPP1 and ENPP3-5 hydrolyze nucleotides, while ENPP2 and ENPP6-7 have evolved as phospholipases due to adaptations in the catalytic domain (Borza et al., 2022). ENPP1, previously known as plasma cell membrane glycoprotein-1 (PC-1), is the most extensively studied family member that catalyzes nucleotides to generate PPi, thereby inhibiting mineralization processes (Terkeltaub, 2006; Roberts et al., 2019). Given that the balance between Pi and PPi regulates mineralization, ENPP1 has garnered significant pathological interest in mineralization disorders and soft tissue calcification. This interest stems from the fact that ENPP is the only family member with various disease-associated loss-of-function mutations (Nitschke et al., 2011; Jansen et al., 2012; Kato et al., 2012; Onyedibe et al., 2019). Studies have demonstrated that *ENPP1*-null mice exhibit hypermineralization abnormalities, including cartilage calcification in osteoarthritis and ossification of the spinal ligament in spinal hyperostosis (Sakamoto et al., 1994; Okawa et al., 1998; Rutsch et al., 2001).

To elucidate the mechanism underlying PDL fibroblastic differentiation, our focus centered on identifying cell surface or extracellular matrix (ECM) components specifically expressed during TGF-β1-induced fibroblastic differentiation of hPDLSCs. Consequently, we developed a set of monoclonal antibodies that recognize the cell surface and ECM components of PDL fibroblasts. Notably, one of these antibodies targets ENPP1, shedding light on its role in PDL fibroblast differentiation. Additionally, in our recent transcriptomic analysis, we noted specific ENPP1 expression in PDL fibroblasts, contrasting with ENPP2 expression in cementoblasts (Mun et al., 2022). As ENPP2 expression was entirely suppressed in PDL fibroblasts, we aimed to elucidate the relationship between ENPP2 and ENPP1 function in TGF-β1-induced PDL fibroblastic differentiation.

## Materials and methods

### Cell culture and treatment

For the culture of human periodontal ligament stem cells (hPDLSCs), third molars were obtained from adult patients (19–23 years of age) under guidelines approved by the IRB of the Dankook University (DKU NON 2020-008) with approval of patients visiting DKU Dental Hospital. PDL tissues were digested by 4 mg/mL dispase (Sigma-Aldrich) and 3 mg/mL collagenase type I (Millipore) for 1 h at 37°C. Single cell suspension isolated from tissue was incubated with α-MEM (Hyclone) containing 20% fetal bovine serum (FBS, Hyclone) and antibiotics (Lonza) at 37°C in 5% CO_2_. hPDLSCs were characterized by immunophenotyping using antibodies against representative mesenchymal stem cell surface antigens (Supplementary Figure S1). For PDL fibroblastic differentiation, hPDLSCs were cultured in 6-well plates at a density of 2 × 10^4^ cells per well in α-MEM containing 5% FBS, and 10 ng/mL TGF-β1 (Sino Biological) was treated for 9 days. For osteo/cementoblastic differentiation, 10 μM SB431542 (TOCRIS) and 100 ng/mL BMP7 (Prospec) were cotreated for 9 days. Pyrophosphate (PPi) and lysophosphatidic acid (LPA) were treated at 100 μM and 20 μM, respectively. All the cytokines and chemicals were replaced with new medium every 2 days. For mineral formation, cells were incubated in media containing the mineralization additives (OM), which consist of 100 μM ascorbic acid, 100 nM dexamethasone, and 5 mM β-glycerophosphate for 21 days. HeLa cells were cultured with DMEM (Hyclone) containing 10% FBS at 37°C in 5% CO_2_.

### Immunophenotyping and flow cytometric analysis

Cells dissociated by enzyme-free dissociation solution (Millipore) were incubated with proper antibodies in PBS containing 1% BSA on ice, followed by treatment with FITC-conjugated anti-mouse IgG (1:100, Santa Cruz) as the secondary antibody. Cells were analyzed by flow cytometry in FACSCalibur™ (BD Biosciences). Antibody binding affinity was quantified by using WinMDI program.

### Quantitative real time polymerase chain reaction (qRT-PCR)

Total RNA was extracted using the AccuPrep^®^ Universal RNA Extraction Kit (Bioneer) following the experimental protocol provided by the manufacturer. cDNA was synthesized by using the ReverTra Ace™ qPCR RT kit (Toyobo). The qRT-PCR was performed by using iTaq™ Universal SYBR Green Supermix (Bio-Rad). Used primers are listed in Supplementary Table S1. The qRT-PCR reactions were carried out using the StepOnePlus Real-Time PCR system (Thermo Fisher Scientific). The cycling parameters of qPCR were followed: one cycle for 30 s at 95°C for denaturation and 40 cycles for 15 s at 95°C and 1 min at 60°C for annealing. The melting curve analysis was conducted by gradually increasing the temperature from 65°C to 95°C in 0.5°C increments. The *GAPDH* was used as an internal control to normalize the variability in target gene expression. The relative quantity (ΔCq) for each gene was calculated from the threshold cycles obtained by using the formula ΔCq = 2 ^ [Cq (Min) - Cq (Sample)]. Cq (Min) represents the lowest average threshold cycle among the samples for the gene of interest, and Cq (Sample) represents the average threshold cycle for the sample. The normalized expression level (ΔΔCq) is the relative amount of each gene normalized to the quantity of *GAPDH* gene. The calculation for normalized expression was followed by formula ΔΔCq = ΔCq (Sample)/ΔCq (Ref). For statistical analysis, unpaired Student t-test was applied using GraphPad Prism software. The values are presented as the mean values ±SD. All experiments were repeated 3 times independently, and the statistical significance value was set at *p* < 0.05 and represented as ***, *p* < 0.001; **, *p* < 0.01; *, *p* < 0.05.

### Immunocytochemistry

To detect subcellular localization of the antigenic molecule, the immunostaining of cells was performed. Cells on the cover slips were washed with cold PBS and then fixed in 4% paraformaldehyde at 4°C for 1 h. After blocking, cells were incubated with the primary antibody diluted 1:200 in 5% horse serum at 4°C for 16 h. FITC-conjugated anti-rabbit IgG or FITC-conjugated anti-mouse IgG (Jackson Immuno Research) was added as a secondary antibody. Phalloidin-FITC reagent (Abcam) and 4,6-diamidino-2-phenylindole (DAPI, Sigma) were used for detection of cytoskeletal distribution and nuclei, respectively. Samples were observed by a confocal microscope LSM700 (Carl Zeiss).

### Immunoprecipitation and Western blot analysis

Cells were lysed using 1% NP-40 buffer (20 mM Tris-HCl, pH 8.0, 150 mM NaCl, 2 mM EGTA, 2 mM EDTA, 1% NP-40, phosphatase/protease inhibitors). For immunoprecipitation, cell extract was incubated with the primary antibody for 16 h at 4°C, followed by incubation with Protein G-agarose (Santa Cruz) for 3 h at 4°C. The immunoprecipitants were separated on SDS-PAGE, transferred to a PVDF membrane (Millipore). For Western blot analysis, and then probed with the HRP conjugated secondary antibody in blocking buffer at room temperature for 1 h. For identification of surface antigens, intact cells were labeled by EZ-Link Sulfo-NHSLC-Biotin (Thermo Scientific). Biotin-labeled cell extract was used for immunoprecipitation and immunoprecipitates were detected by streptavidin-HRP (GE Healthcare). Signals were visualized by using ECL Western Blotting Detection Kit (GE healthcare) under X-ray film.

### Immunohistochemistry

The tooth root part of the human third molar was fixed in 4% paraformaldehyde (Sigma), followed by washing with tap water. The tooth pieces were then decalcified by immersion in RapidCal solution (BBC Biochemical) at room temperature and subsequently dehydrated in various concentrations of ethanol. Subsequently, the teeth were cleared by incubating in xylene. The transparent teeth were embedded in paraffin (Leica) to create paraffin blocks. The paraffin blocks sectioned into 6-μm thickness using a microtome (RM2255, Leica) were deparaffinized by incubating in xylene and in various concentrations of ethanol and were rehydrated. For antigen retrieval, 0.05% trypsin was applied, followed by treatment with 3% H_2_O_2_. After blocking, the tissue sections were incubated with the primary antibody and subsequentially the biotinylated anti-mouse IgG (Vector Laboratories). For detection of signals, tissue sections were treated with VECTASTAIN ABC Reagent (Vector Labs) and diaminobenzidine (DAB) (Vector Labs) until the desired signal was developed according to the manufacturer’s instructions. Sample slides were mounted with Eukitt quick-harder mounting medium (Sigma) and observed using Eclipse 80i upright microscope (Nikon).

### cDNA constructs and ectopic expression

Full-length cDNAs of *ENPP1* and *ENPP2* were subcloned in pcDNA3.1 (+). All the clones were tagged by DYKDDDDK in C-terminus. The flanking sequences of the cloning site and full ORF sequences were confirmed by sequence analysis. For ectopic expression of these construction, cells were transfected with DNAs using Lipofectamine^®^ 2000 (Thermo Fisher Scientific) according to the manual provided by the manufacturer. During the PDL fibroblastic differentiation for 9 days, DNA transfection was carried out repeatedly in every 2 days.

### Gene silencing

Small interfering RNAs (siRNAs) for *ENPP1 and ENPP2* were designed and synthesized by Bioneer Corporation. 80,000 cells were used for knock-down and transfected with 1.5 μg of siRNA by using Lipofectamine^®^ RNAiMAX (Thermo Fisher Scientific) according to the manual provided by the manufacturer. Media were changed after incubation with siRNA for 5 h. During the PDL fibroblastic differentiation for 9 days, siRNA transfection was carried out repeatedly in every 2 days.

### Cell proliferation assay

Cell growth rate was determined using the Cell Counting Kit-8 following the manufacturer’s protocols (MedChemExpress). Cells were seeded in a 96-well plate at a density of 10^4^-10^5^ cells/well in 100 μL of culture medium with or without siRNA constructs to be tested, and incubated for an appropriate length of time (e.g., 3, 5, 7 or 9 days) in the incubator. After incubation, 10 μL of CCK-8 solution were added to each well of the plate. The absorbance at 450 nm were measured using a microplate reader.

## Results

### The antigenic molecule recognized by the novel anti-PDL13 antibody is significantly upregulated in TGF-β1-induced PDL fibroblasts

**FIGURE 1:**
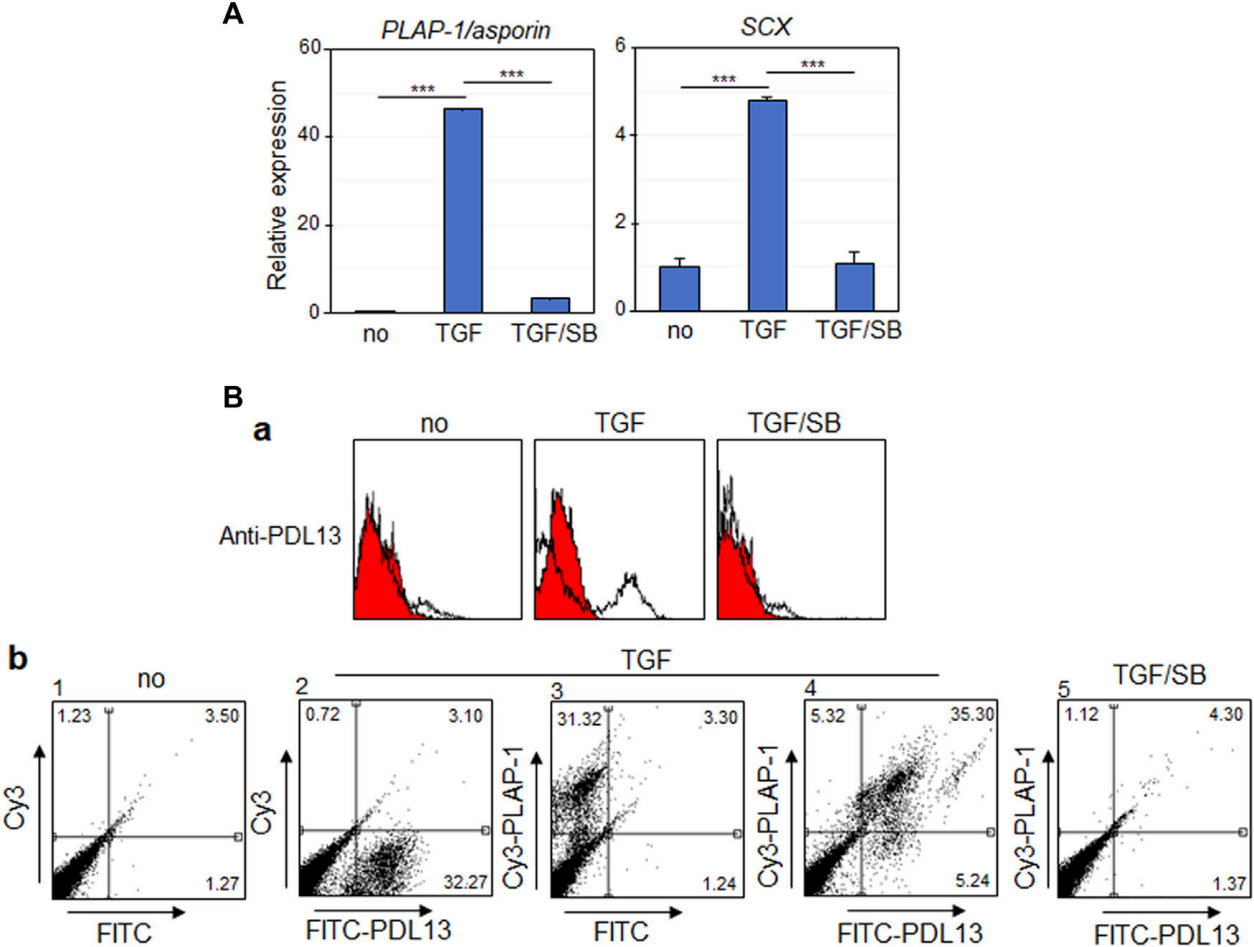
The antigenic molecule recognized by anti-PDL13 antibody is highly increased in TGF-β1-induced PDL fibroblastic differentiation of human periodontal ligament cells (hPDLCs). **(A)** TGF-β1 treatment promotes fibroblastic differentiation of hPDLCs. The primary cultured hPDLCs were treated with 10 ng/mL TGF-β1 (TGF) for 9 days for induction of fibroblastic differentiation. 10 μM SB431542 was treated together with TGF-β1 (TGF/SB). The transcriptional expression of representative markers in cells were analyzed by qRT-PCR. **(B)** Anti-PDL13 antibody recognizes a PDL fibroblast-specific antigen. Histograms **(a)** and dot plots **(b)** in flow cytometry showed the fluorescence intensity of bound antibodies in undifferentiated hPDLCs (*no*) and hPDLCs treated with TGF-β1 and/or SB431542 (*TGF* and *TGF/SB*). In **(b)**, for detection of anti-PDL13-positive cells, Fluorescein isothiocyanate (FITC)-labeled anti-Mouse IgG secondary antibody was used (FITC-PDL13). For detection of PDL fibroblasts, anti-PLAP-1/asporin antibody and Cyanine3 (Cy3)-labeled anti-Rabbit IgG secondary antibody (Cy3-PLAP-1).

When hPDLSCs were exposed to 10 ng/mL of TGF-β1 for 9 days, the expression levels of two representative PDL fibroblastic markers, such as periodontal ligament-associated protein-1 (*PLAP-1/asporin*) and scleraxis (*SCX*), were elevated (Figure 1A, TGF). However, their expressions were significantly reduced upon treatment with SB431542, a TGF-β type I receptor inhibitor (Figure 1A, TGF/SB). Consequently, hPDLSCs were induced to differentiate into fibroblastic cells by TGF-β1 treatment and were utilized for further experimentation as PDL fibroblasts. Previously, we developed a panel of monoclonal antibodies targeting the membrane/ECM molecules expressed in TGF-β1-induced PDL fibroblasts. Among these antibodies, the novel IgG-type antibody, anti-PDL13, was employed in this study to identify PDL fibroblast-specific antigens. Flow cytometric analysis revealed a significantly increased cell-binding affinity of the anti-PDL13 antibody in PDL fibroblasts (Figure 1B, TGF in a). However, this antibody binding was not sustained, when the TGF-β1 signaling pathway was inhibited by SB431542 treatment (Figure 1B, TGF/SB in a). Certainly, PDL fibroblasts exhibited dual positivity for anti-PLAP-1/asporin and anti-PDL13 antibodies. However, the simultaneous disappearance of cell-binding affinity for these antibodies occurred after SB431542 treatment (Figure 1B, panels four and five in b). These findings imply that the antigen recognized by the anti-PDL13 antibody might serve as a specific marker of TGF-β1-induced PDL fibroblasts.

### The antigenic molecule recognized by a novel anti-PDL13 antibody corresponds to ectonucleotide pyrophosphatase/phosphodiesterase 1 (ENPP1)

cDNA sequencing was conducted using total RNA from a hybridoma-producing the anti-PDL13 antibody. Antibody similarity analysis based on the IMGT/V-QUEST database was employed to define the complementarity-determining regions (CDRs) of anti-PDL13 antibody (Brochet et al., 2008; Ning et al., 2012). The amino acid sequences around the variable chains of the CDRs were well conserved (Supplementary Figure S2). The V and J segments of the heavy chain shared 94.44% and 59.09% similarity with Musmus IGHV1S135*01F and Musmus IGHJ3*01F, respectively. The V and J segments of the light chain shared 95.70% and 56.41% similarity with Musmus IGKV19-93*01F and Musmus IGKJ2*01F, respectively (Supplementary Figure S2). These findings indicate that the anti-PDL13 antibody is a novel antibody belonging to the IgG1 and IGκ light chain subgroups.

**FIGURE 2:**
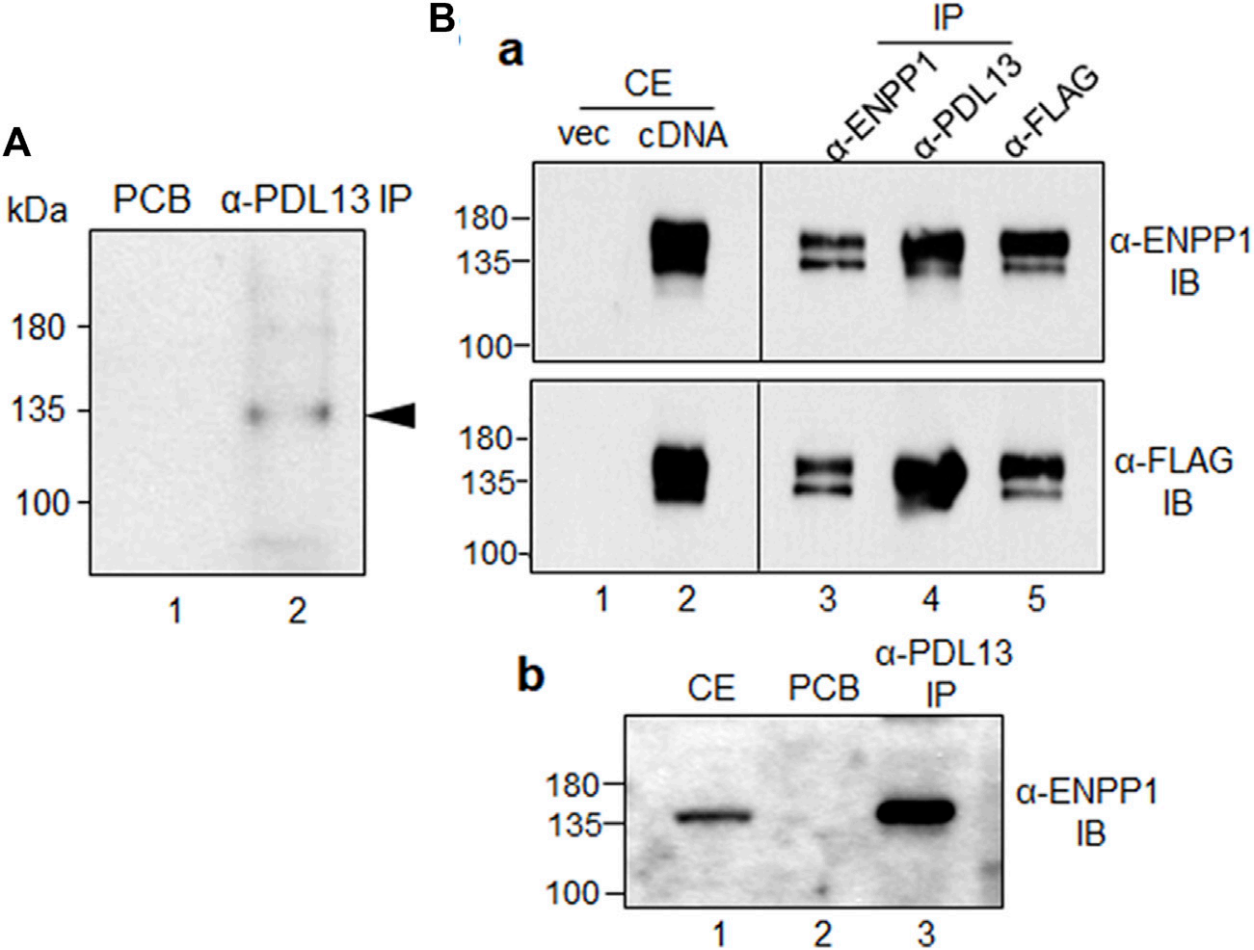
The antigenic molecule recognized by anti-PDL13 antibody is ectonucleotide pyrophosphatase/phosphodiesterase 1 (ENPP1). **(A)** Identification of the antigenic molecule recognized by anti-PDL13 antibody. Intact PDL fibroblasts were labeled with biotin and used for immunoprecipitation with anti-PDL13 antibody. Lane 1, pre-cleared agarose bead (PCB); lane 2, anti-PDL13 immunoprecipitates (IP). **(B)** Cross-reactivity between anti-PDL13 antibody and a commercial anti-ENPP1 antibody in HeLa cells expressing *ENPP1* cDNA **(a)** and in TGF-β1-induced PDL fibroblasts **(b)**. Full length cDNA construct tagged by FLAG was expressed in HeLa cells and total cell extract (CE) detected by anti-FLAG and anti-ENPP1 antibodies. Anti-PDL13 immunoprecipitates (*α-PDL13 IP*) recognized by immunobinding assay (IB) with both anti-FLAG (*α-FLAG IB*) and anti-ENPP1 (*α-ENPP1 IB*) antibodies.

To detect the antigen recognized by the anti-PDL13 antibody, immunoprecipitation was performed using cell extracts from biotin-labeled PDL fibroblasts. Protein bands of approximately 130 kDa were strongly detected in the anti-PDL13 immunoprecipitates using a streptavidin-HRP-conjugated secondary antibody (Figure 2A, lane 2). Mass spectrometry analysis of the protein band cut from the gel identified the antigen as ectonucleotide pyrophosphatase/phosphodiesterase 1 (ENPP1), also referred to as plasma-cell membrane glycoprotein (PC-1). The full-length human *ENPP1* cDNA construct was cloned into a mammalian FLAG-tagged expression vector and ectopically expressed in HeLa cells. Almost no endogenous ENPP1 protein was detected in HeLa cells (Figure 2B, lane one in the upper panel in a), suggesting that this protein is rarely expressed in HeLa cells. Ectopically expressed ENPP1 was detected using a commercially available anti-ENPP1 antibody (Santa Cruz, raised against amino acids 9-36) and an anti-FLAG antibody (Figure 2B, lanes two in a). Immunoprecipitates obtained with the anti-PDL13 antibody were strongly recognized by both commercial anti-ENPP1 antibody and the anti-FLAG antibody (Figure 2B, lane four in a). Immunoprecipitates obtained with the anti-FLAG antibody were strongly detected by the anti-ENPP1 antibody (Figure 2B, lane five in the upper panel in a). Despite efficient immunoprecipitation, our novel anti-PDL13 antibody was not suitable for Western blot analysis of denatured proteins. Furthermore, immunoprecipitates generated with the anti-PDL13 antibody from cell extracts of TGF-β1-induced PDL fibroblasts were robustly detected by a commercial anti-ENPP1 antibody (Figure 2B, lane three in b). These findings confirm the identity of the PDL13 antigen as ENPP1.

### ENPP1 is upregulated in TGF-β1-induced PDL fibroblasts and accumulated in the periodontal ligament of human tooth roots

**FIGURE 3:**
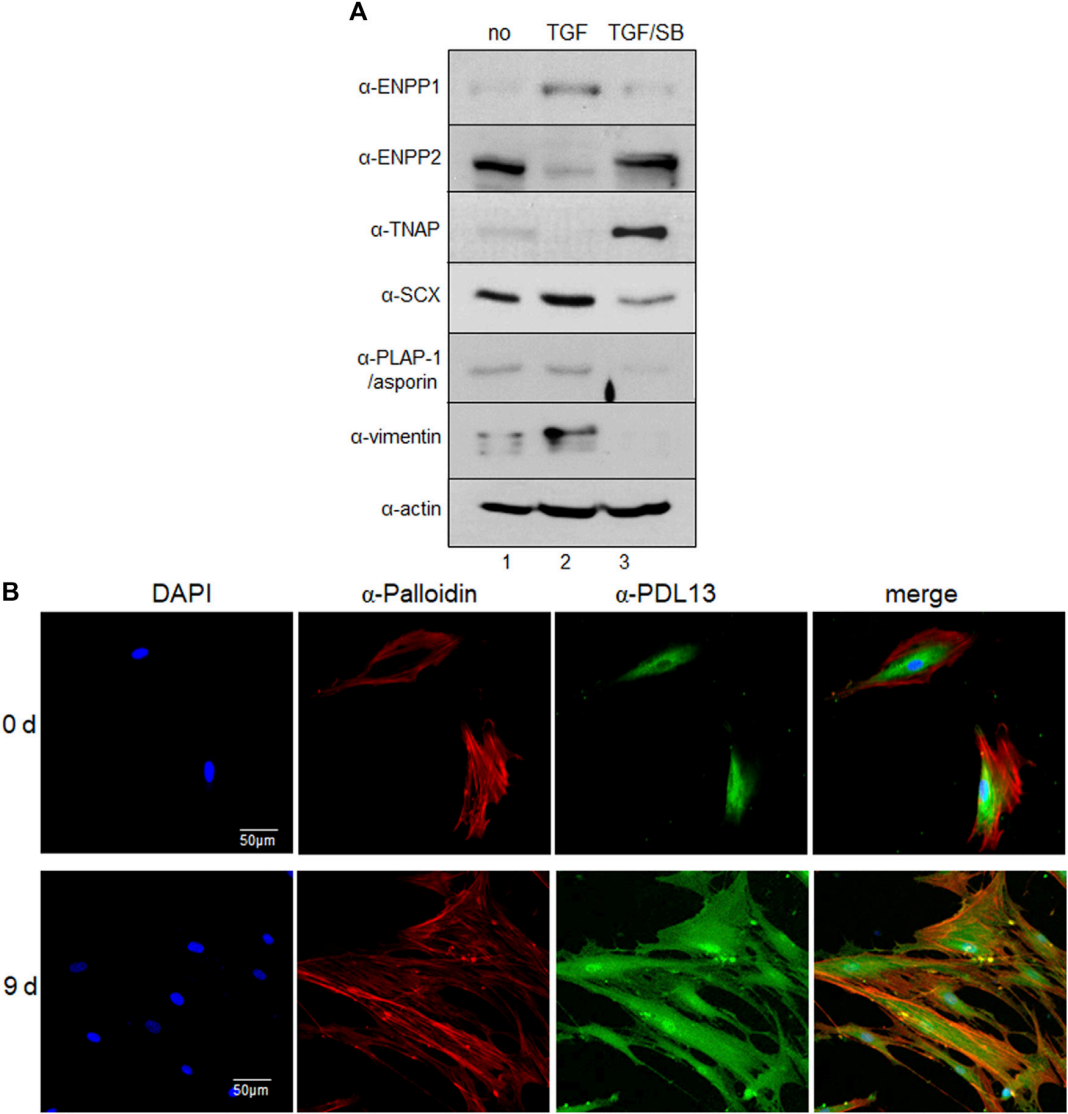
ENPP1 protein is upregulated in TGF-β1-induced PDL fibroblasts and accumulated in human periodontal ligament. **(A)** The expression level of representative proteins in hPDLCs. hPDLCs were treated with TGF-β1 (TGF) and/or SB431542 (SB) and used for preparation of total cell extract. Lane 1, undifferentiated hPDLCs; lane 2, TGF-β1-induced PDL fibroblasts; lane 3, hPDLCs treated with SB431542 for inhibition of TGF-β1 function. **(B)** Subcellular localization of ENPP1/PDL13 was examined by immunocytochemistry in PDL fibroblasts. ENPP1/PDL13 was detected by anti-PDL13 antibody and FITC-labeled secondary antibody, and the cell boundary was detected by Cy3-palloidin. Nucleus region in cells was stained by 4',6-diamidino-2-phenylindole (DAPI).

The amount of ENPP1 increased in PDL fibroblasts but decreased under the condition of inhibition of the TGF-β1 signaling pathway (Figure 3A, lanes two and three in α-ENPP1). Among the ENPPs family members, ENPP2 contains a unique hydrophobic pocket next to the catalytic phosphodiesterase domain due to the deletion of 18 residues compared to ENPP1 (Kato et al., 2012; Borza et al., 2022). Interestingly, the expression of ENPP2 in hPDLSCs was totally blocked by TGF-β1 treatment, suggesting that ENPP1 and ENPP2 were produced in an opposite manner according to stimulation or inhibition of TGF-β1 signaling pathway in hPDLSCs (Figure 3A, α-ENPP1 and α-ENPP2). Tissue-non-specific alkaline phosphatase (TNAP) functions by hydrolyzing extracellular pyrophosphate (PPi), which is a potent mineralization inhibitor generated by ENPP1, thereby aiding in skeletal and dental mineralization process. TNAP and ENPP1 acts as antagonistic regulators of bone mineralization (Hessle et al., 2002; Foster et al., 2012). Specifically, TGF-β1 stimulation resulted in increased ENPP1 expression, while inhibition of the TGF-β1 signaling pathway in PDL fibroblasts notably elevated TNAP expression. However, TNAP expression was not prominently observed in undifferentiated hPDLCs (Figure 3A, α-TNAP). The increased expression levels of representative PDL fibroblastic markers in TGF-β1-induced PDL fibroblasts were diminished by SB431542 treatment (Figure 3A, α-SCX, α-PLAP-1/asporin, and α-vimentin). ENPP1/PDL13 exhibited accumulation around the nucleus in undifferentiated hPDLSCs and migrated throughout the cell surface and cell boundary in TGF-β1-induced PDL fibroblasts (Figure 3B). Immunohistochemistry demonstrated abundant presence of ENPP1/PDL13 in the PDL region between the cementum and alveolar bone in human teeth (Supplementary Figure S3). Consistently, PLAP-1/asporin, a PDL-specific marker, exhibited strong expression in the PDL region (Supplementary Figure S3).

### Ectopic expression of ENPP1 enhances the efficiency of PDL fibroblastic differentiation while suppressing mineralization

**FIGURE 4:**
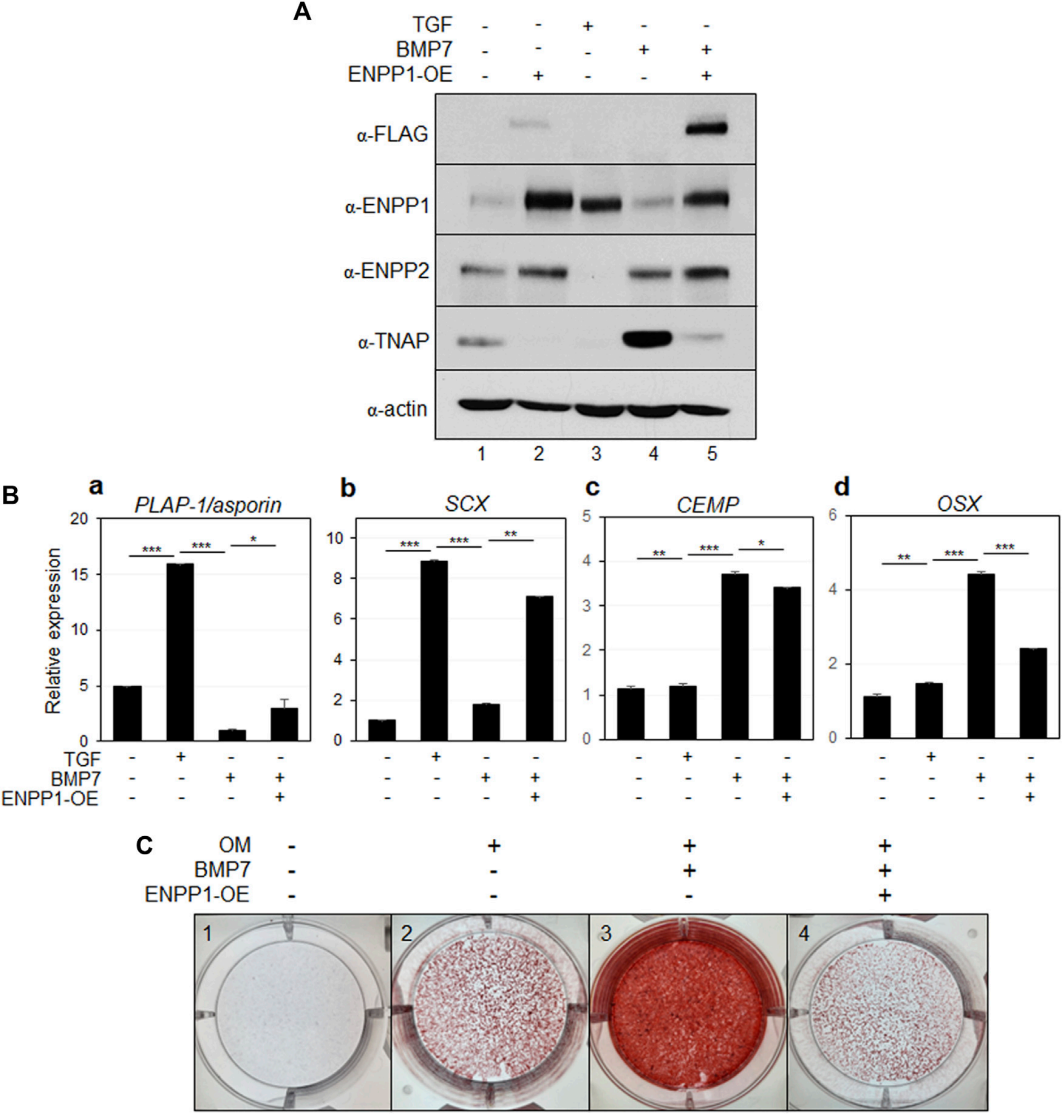
Ectopic expression of ENPP1 inhibits osteo/cementoblastic differentiation and mineralization. **(A)** The expression level of representative proteins in hPDLCs. hPDLCs were treated with cytokines (TGF or BMP7) and transfected with cDNA construct for *ENPP1* overexpression (ENPP1-OE). Total cell extract were prepared and used for immunobinding. **(B)** Transcriptional expression of the representative differentiation markers. Lane 1, undifferentiated hPDLCs; lane 2, hPDLCs treated with TGF-β1; lane 3, hPDLCs treated with BMP7; lane 4, hPDLCs treated with BMP7 and *ENPP1* cDNA (ENPP1-OE). **(C)** Mineralization was inhibited by *ENPP1* overexpression (ENPP1-OE). hPDLCs introduced with *ENPP1* cDNA were incubated in osteoinduction media (OM) for 21 days. Mineralization was analyzed by alizarin staining as described in Materials and methods.

To explore the function of ENPP1 in periodontal ligament differentiation, we introduced full-length ENPP1 protein into undifferentiated hPDLSCs through ectopic overexpression. The ectopic *ENPP1* cDNA was tagged with a FLAG epitope, facilitating its detection using both anti-FLAG and commercial anti-ENPP1 antibodies (Figure 4A, lane four in α-FLAG and α-ENPP1). BMP7, a potent bone-inducing factor, served as a stimulus for cementoblastic differentiation (Hakki et al., 2010; Torii et al., 2016; Choi et al., 2023). In contrast to PDL fibroblasts differentiated via TGF-β1 treatment, ENPP1 expression was not detected in hPDLCs treated with BMP7, whereas TNAP expression significantly increased (Figure 4A, lane three in α-ENPP1 and α-TNAP). Intriguingly, upon overexpression of ENPP1 in BMP7-treated hPDLSCs, the heightened TNAP was attenuated (Figure 4A, lanes three and four in α-TNAP). ENPP2 expression remained relatively stable across all conditions except TGF-β1-induced PDL fibroblasts. Notably, ENPP1 overexpression in BMP7 treatment did not alter ENPP2 expression level (Figure 4A, lanes three and four in α-ENPP2).

Additionally, transcriptional expression of the representative PDL fibroblastic markers were increased in TGF-β1-induced fibroblasts but decreased in BMP7 treated hPDLSCs (Figure 4Ba, b). However, ENPP1 overexpression in hPDLSCs treated with BMP7 resulted in the upregulation of PDL fibroblast markers (Figure 4Ba, b). BMP7 treatment notably induced the expression of representative osteo/cementoblast-specific markers, including cementum matrix protein (*CEMP*) and osterix (*OSX*). However, overexpression of ENPP1 resulted in reduced expression of *OSX* in hPDLCs (Figure 4Bc, d). These findings indicate that elevated ENPP1 levels inhibited the osteo/cementoblastic differentiation induced by BMP7 and instead promoted fibroblastic activation. Furthermore, beyond cyto-differentiation, the impact of ENPP1 overexpression on mineralization was explored.

As observed in Figure 4A, BMP7 treatment led to an increase in TNAP expression (Figure 4A, lane 3), resulting in substantial rise in mineralization level in hPDLSCs, as expected (Figure 4C, panel 3). Remarkably, ectopic ENPP1 dramatically suppressed the mineralization, which was induced by BMP7 (Figure 4C, panel 4). These findings suggest that ectopic ENPP1 effectively inhibits osteo/cementoblastic differentiation mediated by BMP7 and plays a crucial role in maintaining PDL fibroblast differentiation.

**FIGURE 5:**
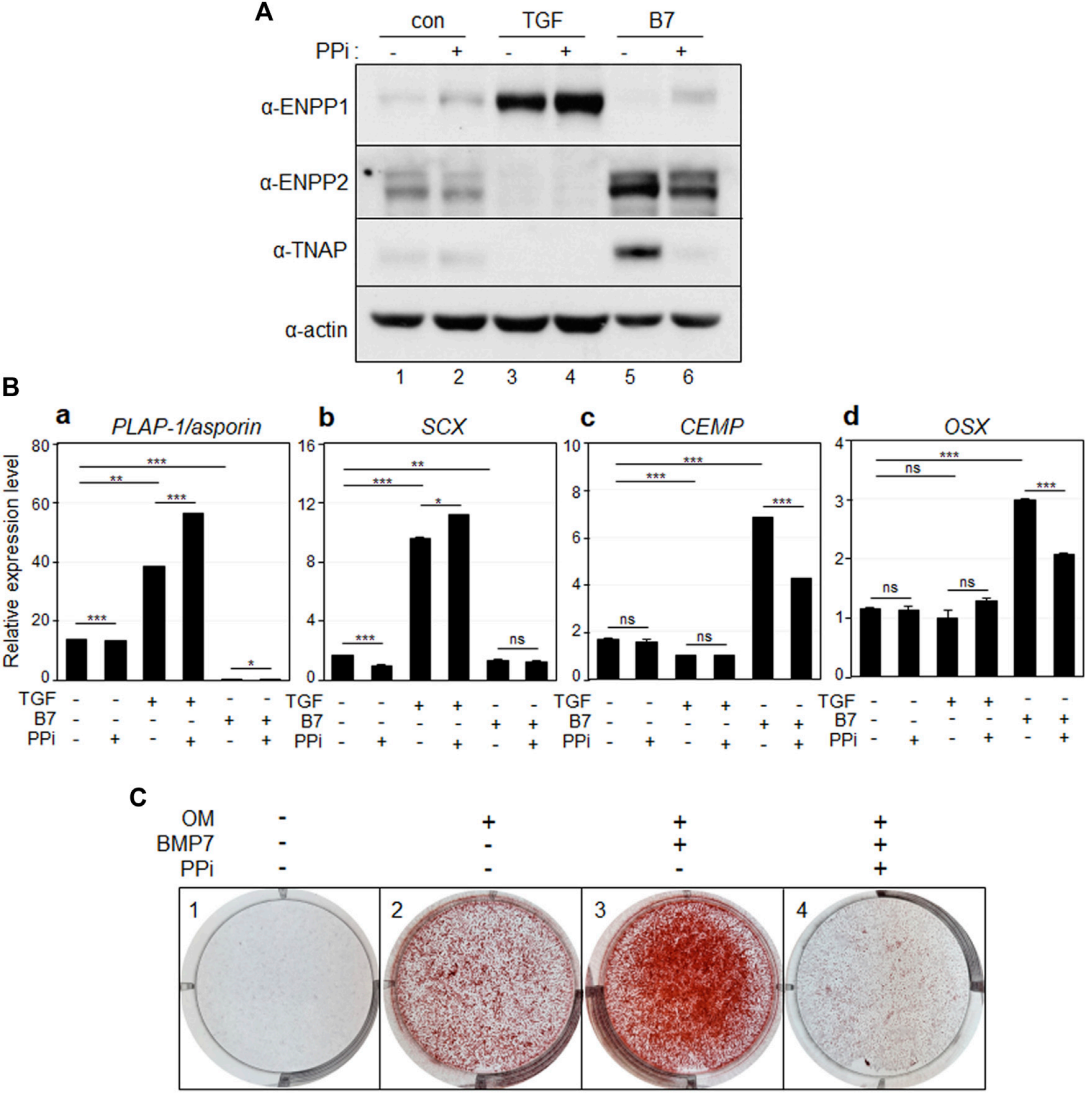
Inorganic pyrophosphate inhibits osteo/cementoblastic differentiation and mineralization. **(A)** The expression level of representative proteins in hPDLCs. hPDLCs were treated with TGF-β1 (TGF) or BMP7 (B7), and/or treated with inorganic pyrophosphate (PPi). Total cell extract were prepared and used for immunobinding. **(B)** Transcriptional expression of the representative differentiation markers. Lane 1, undifferentiated hPDLCs; lane 2, undifferentiated hPDLCs with PPi; lane 3, hPDLCs treated with TGF-β1; lane 4, hPDLCs treated with TGF-β1 and PPi; lane 5, hPDLCs treated with BMP7; lane 6, hPDLCs treated with BMP7 and PPi. **(C)** Mineralization was inhibited by treatment with inorganic pyrophosphate (PPi). hPDLCs treated with BMP7 and/or PPi were incubated in osteoinduction media (OM) for 21 days. Mineralization was analyzed by alizarin staining as described in Materials and Methods.

Inorganic pyrophosphate (PPi) acts as an inhibitor of bone and cartilage mineralization and is generated through the hydrolysis of ATP or GTP, a process catalyzed by ENPP1 (Stefan et al., 2005; Roberts et al., 2019). Upon the addition of PPi, the levels of ENPP1 and ENPP2 remained unchanged (Figure 5A, lanes 2, 4, and 6). Interestingly, the elevated levels of TNAP induced by BMP7 were significantly reduced upon PPi treatment (Figure 5A, lanes five and 6). As expected, the expression of the osteo/cementoblastic markers, *CEMP* and *OSX* was upregulated by BMP7 and downregulated by PPi treatment (Figure 5B). Moreover, mineralization was completely halted by PPi treatment (Figure 5C, panel 4). These findings suggest that the increased catalytic activity of ENPP1 during TGF-β1 treatment downregulates TNAP expression to maintain fibroblastic phenotype by suppressing mineralization in hPDLSCs. Similar to ENPP1 overexpression, PPi appeared to suppress osteo/cementoblastic differentiation, even in the presence of high ENPP2 expression.

### Overexpression of ENPP2 inhibits TGF-β1-induced fibroblast differentiation but does not directly induce mineralization of hPDLCs

It is noteworthy that while ENPP1 was scarcely expressed in undifferentiated PDL cells, the expression of ENPP2, which was not expressed at all during TGF treatment, remained high (Figures 3A, 4A, lane 1). Furthermore, as depicted in Figure 4A, the expression of fibroblastic markers was somewhat suppressed, even when ENPP1 was ectopically expressed in osteo/cementoblastic cells at a similar level as in TGF-β1-induced fibroblasts (Figure 4A, lanes two and four in α-ENPP1). The most significant contrast between these cell types was the quantity of endogenous ENPP2. ENPP2 expression entirely blocked by TGF-β1 was markedly increased by BMP7 regardless of ectopic ENPP1 (Figure 4A, lanes two and four in α-ENPP2). Consequently, it was imperative to explore the impact of ectopic expression of ENPP2 on PDL fibroblastic differentiation by TGF-β1.

**FIGURE 6:**
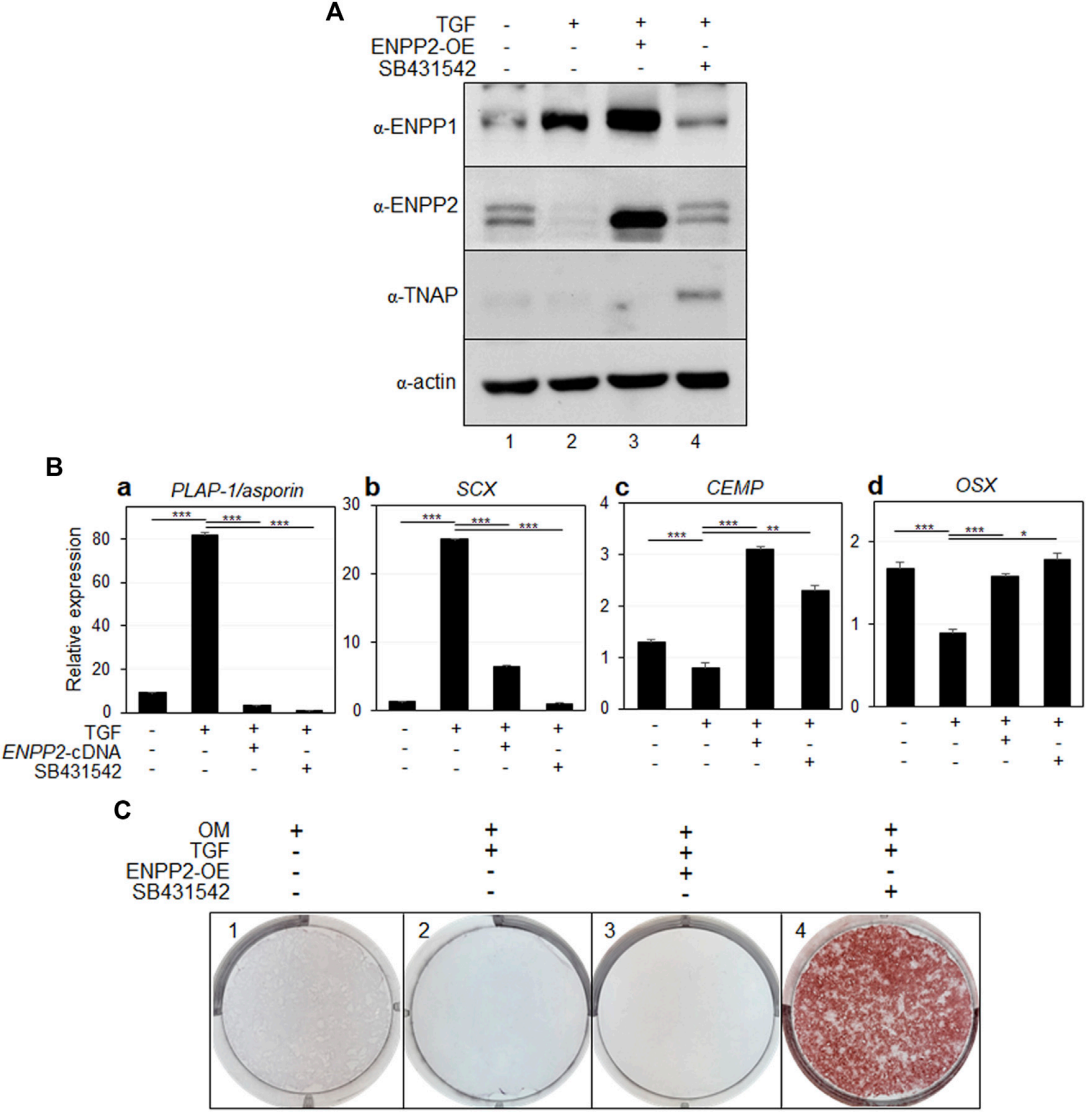
Ectopic expression of ENPP2 inhibits PDL fibroblastic differentiation induced by TGF-β1 but has no effect on mineralization. **(A)** The expression level of representative proteins in hPDLCs. hPDLCs were treated with TGF-β1 (TGF) and/or transfected with *ENPP2* cDNA construct for ENPP2 overexpression (ENPP2-OE). Total cell extract were prepared and used for immunobinding. SB431542 was used for as a control for inhibition of TGF-β1-induced PDL fibroblastic differentiation. Lane 1, undifferentiated hPDLCs; lane 2, hPDLCs treated with TGF-β1; lane 3, hPDLCs treated with TGF-β1 and *ENPP2* cDNA; lane 4, hPDLCs cotreated with TGF-β1 and SB431542. **(B)** Transcriptional expression of the representative differentiation markers. **(C)** Mineralization was not affected by ENPP2 overexpression. For induction of mineralization. cells were incubated in osteoinduction media (OM) for 21 days. Mineralization was analyzed by alizarin staining as described in Materials and methods.

Upon overexpression of ENPP2 in PDL fibroblasts, even in the presence of sufficiently high levels of ENPP1 (Figure 6A, lanes two and three in α-ENPP2 and α-ENPP1), the expression of representative PDL markers significantly decreased (Figure 6B, a and b). Conversely, the expression of osteo/cementoblastic differentiation markers, which were suppressed by TGF-β1 treatment, was instated in cells expressing ectopic ENPP2 (Figure 6Bc, d).

While ENPP2 overexpression effectively inhibited TGF-β1-induced fibroblastic differentiation, it appeared to have no effect on mineralization, which is inhibited by TGF-β1 (Figure 6C, panels two and 3). As a control, we demonstrated that when the TGF-β1 signaling pathway was initially blocked by SB431542 treatment, mineralization inhibited by TGF-β1 was prominently detected (Figure 6C, panel 4).

### Inhibition of ENPP1 reduces the fibroblastic differentiation efficiency of hPDLCs

**FIGURE 7:**
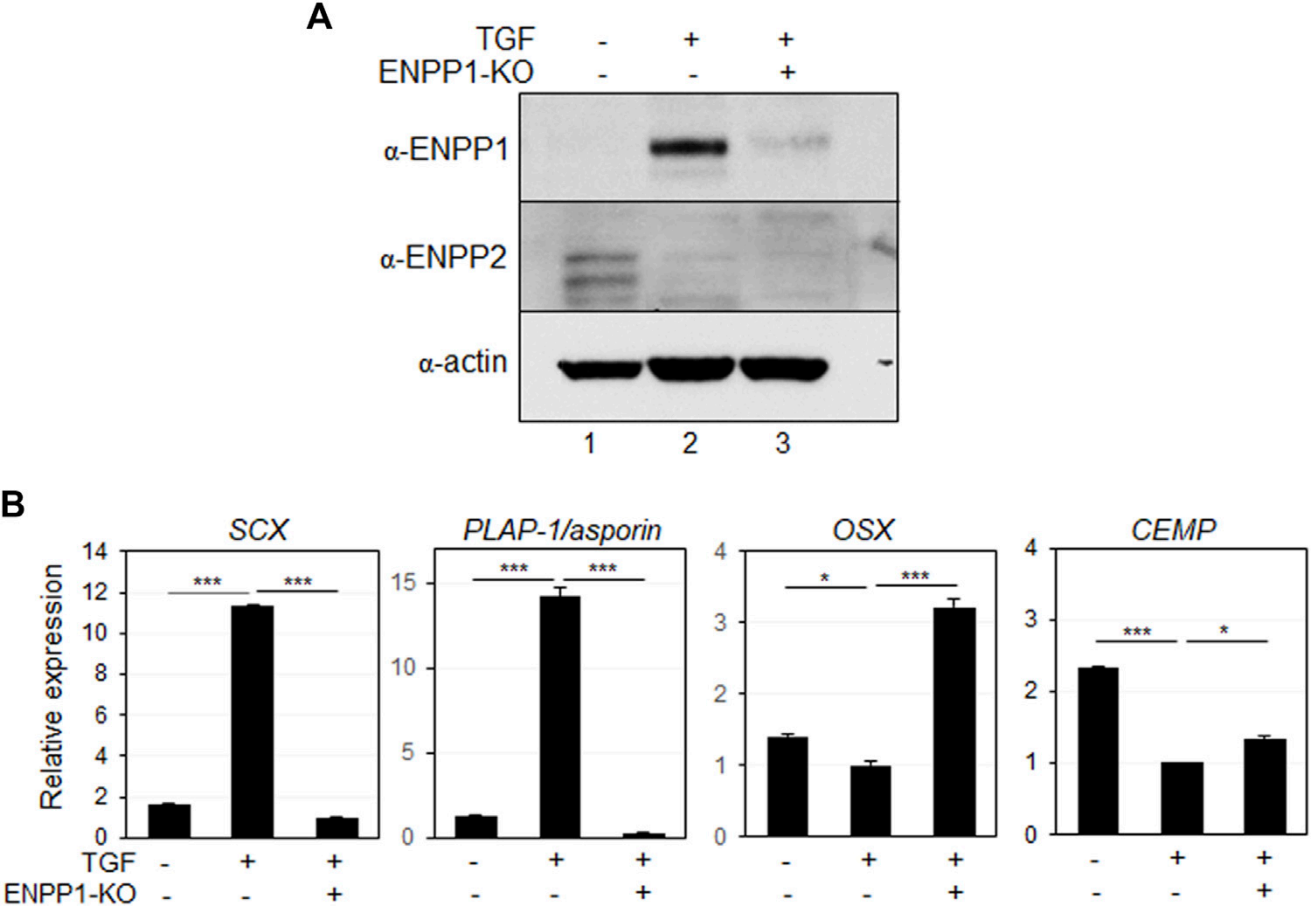
Depletion of ENPP1 reduced the efficiency of TGF-β1-induced PDL fibroblastic differentiation. **(A)** Expression amounts of ENPP1 protein in hPDLCs and PDL fibroblasts were dramatically decreased by introducing siRNA to knock out a gene expression (ENPP1-KO). Lane 1, undifferentiated hPDLCs; lane 2, undifferentiated hPDLCs with *ENPP1* siRNA; lane 3, TGF-β1-induced PDL fibroblasts; lane 4, PDL fibroblasts with siRNA. **(B)** Transcriptional expression of the representative PDL fibroblastic and osteo/cementoblastic markers.

Depleting endogenous ENPP1 using siRNA constructs (Figure 7A, lanes two and 3) resulted in a significant decrease in the expression of *SCX* and *PLAP-1/asporin,* which were initially increased by TGF-β1 treatment, while markers of osteo/cementoblastic differentiation were upregulated (Figure 7B). Additionally, ENPP1 depletion in TGF-β1-induced PDL fibroblasts markedly increased mineralization (Supplementary Figure S4).

**FIGURE 8:**
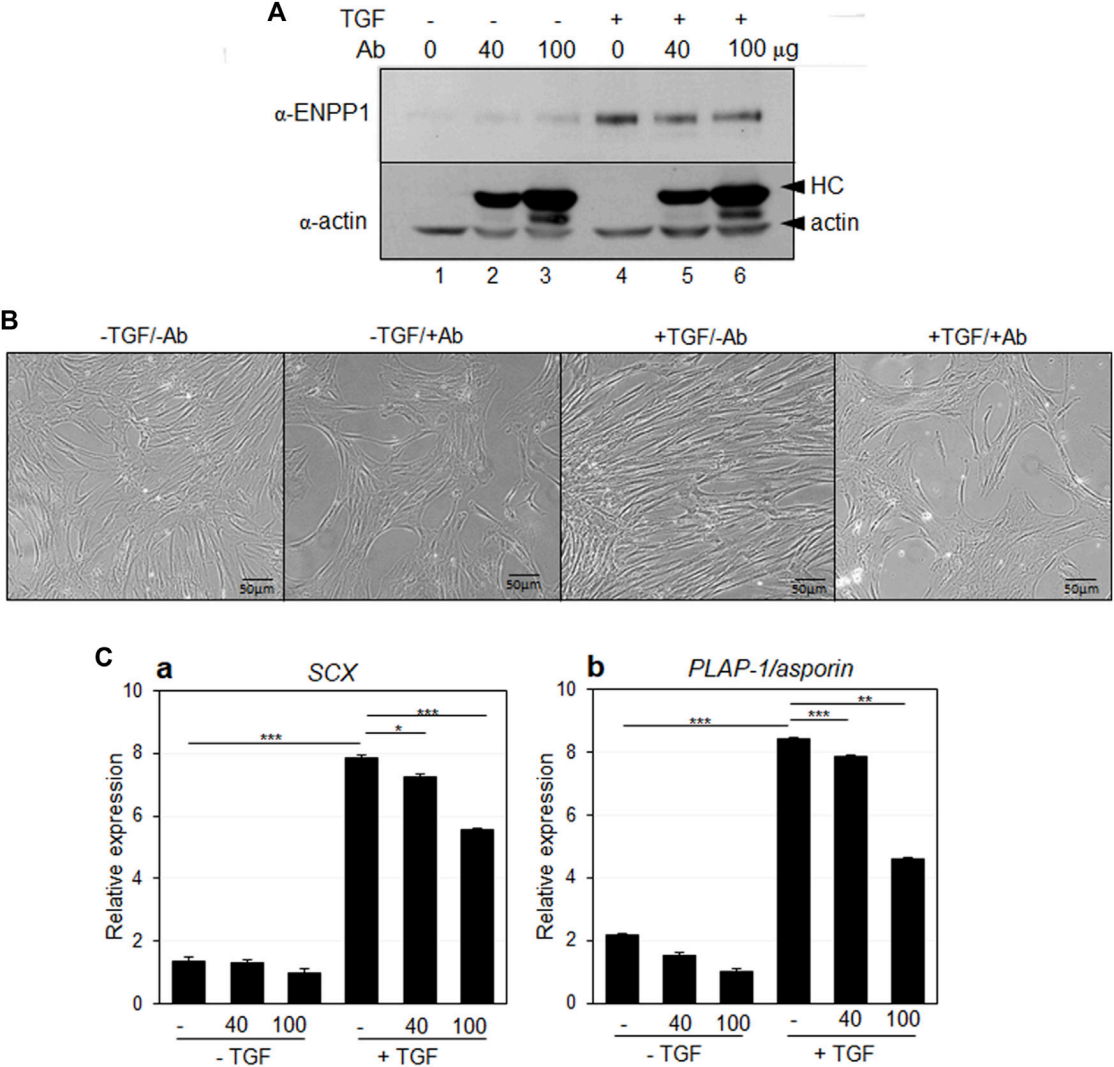
Antibody blocking of ENPP1 by anti-PDL13 antibody. **(A)** Expression of ENPP1 protein in hPDLCs and PDL fibroblasts. Increased ENPP1 by TGF-β1 (TGF) was not changed by treatment with anti-PDL13 antibody (Ab). Lane 1 and 4, no treated cells; lane 2 and 5, cells treated with 40 μg/mL of antibody; lane 3 and 6, cells treated with 100 μg/mL of antibody. Upper arrowhead in α-actin panel indicated heavy chains (HC) of anti-PDL13 antibody, which were internalized in cells. **(B)** Changes in elongated spinous cell morphology by antibody blocking of ENPP1. -TGF/-Ab, hPDLCs; -TGF/+Ab, hPDLCs treated with 40 μg/mL of anti-PDL13 antibody; +TGF/-Ab, TGF-β1-induced PDL fibroblasts; +TGF/+Ab, PDL fibroblasts treated with 50 μg/mL of anti-PDL13 antibody. **(C)** Transcriptional expression of the representative PDL fibroblastic markers. *ns,* not significant; *, *p* < 0.05; **, *p* < 0.01; ***, *p* < 0.001.

In addition to gene depletion via siRNA, the anti-PDL13 antibody, which specifically binds to the cell surface and accumulates intracellularly, seemed to effectively block almost all endogenous ENPP1 when large amounts of the antibody are added to the medium. Antibody treatment did not impact endogenous ENPP1 expression in TGF-β1-induced PDL fibroblasts (Figure 8A, lanes 4-6). Remarkably, hPDLSCs treated with TGF-β1 for 7 days displayed spinous elongated cell processes (Figure 8B, +TGF/-Ab). However, upon inhibition of ENPP1 in PDL fibroblasts through antibody blockade, the cells exhibited a less elongated appearance, resembling the control cells without TGF-β1 treatment (Figure 8B, -TGF). After 7 days of differentiation, the expression of *SCX* and *PLAP-1/asporin* decreased proportionally as the concentration of the antibody increased (Figure 8Ca, b). These findings strongly suggest that ENPP1 inhibition downregulates the fibroblastic differentiation of hPDLSCs.

### Suppression of both ENPP1 and ENPP2 expressions results in decreased cell viability

**FIGURE 9:**
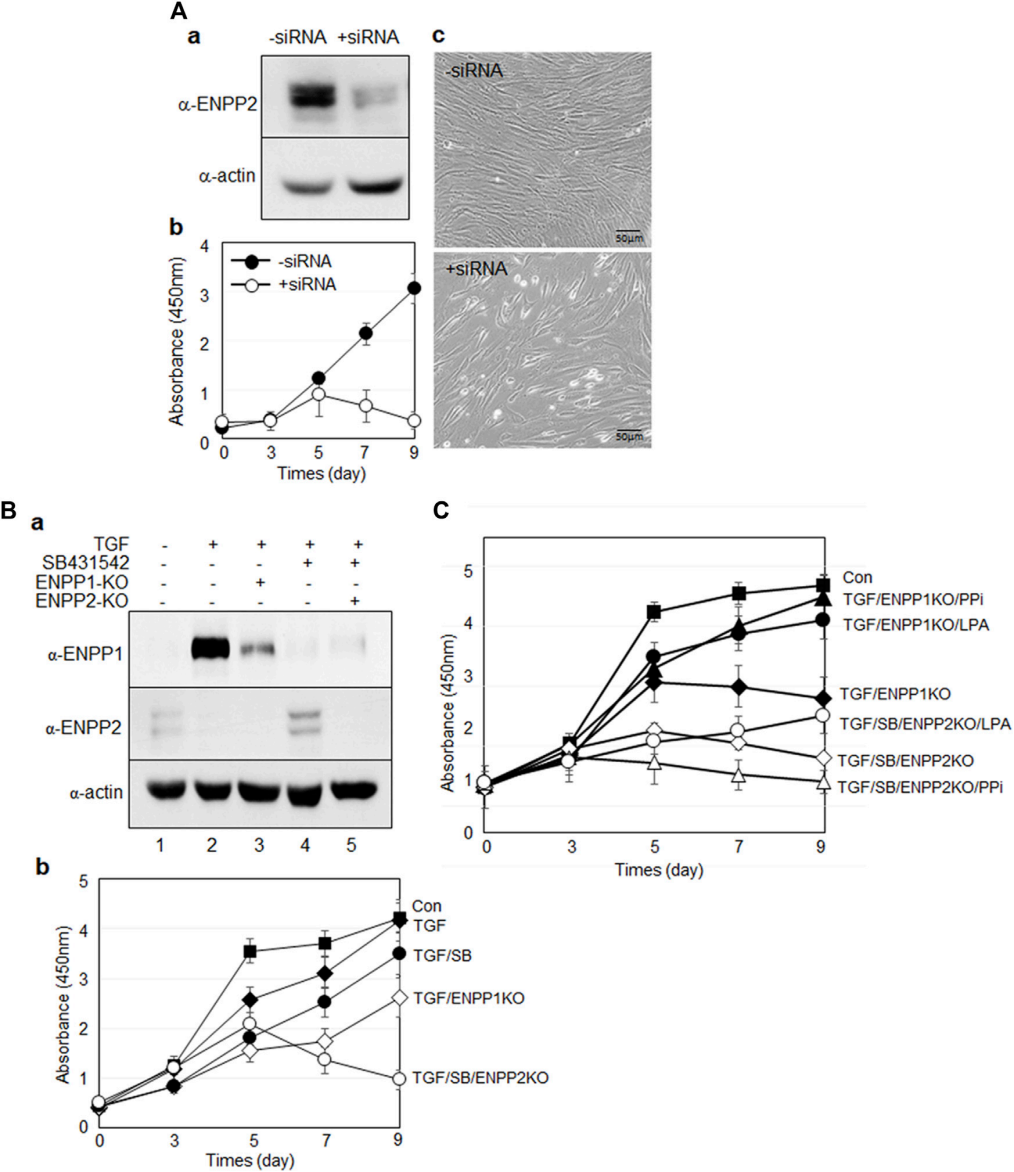
Suppression of ENPP1 and/or ENPP2 expression in undifferentiated and differentiated hPDLSCs. **(A)** Depletion of ENPP2 in undifferentiated hPDLCs. **(a)**, Expression amounts of ENPP2; **(b)**, cell viability assay; c, cell morphology. In b, cell proliferation was analyzed using CCK-8 as described in Materials and Methods. Filled and open circle indicated undifferentiated hPDLCs and depletion of ENPP2, respectively. **(B)** Cell viability of cells depleted of ENPP1 in the absence of ENPP2 and *vice versa.* **(a)**, Expression amounts of ENPP1 and ENPP2; (**b)**, cell viability assay. In **(a)**, lane 1∼5 indicated undifferentiated hPDLCs, TGF-β1-induced PDL fibroblasts, PDL fibroblasts with depleted ENPP1, PDL fibroblasts treated with SB431542, and PDL fibroblasts treated with SB431542 and *ENPP2* siRNA (ENPP2-KO) in that order. **(C)** Effect of the major enzyme products of ENPP1 and ENPP2 on cell survival.

As ENPP2 was expressed even in undifferentiated hPDLSCs (Figures 3A, 4A), we initially investigated the impact of ENPP2 depletion using siRNA. Depletion of ENPP2 in undifferentiated hPDLSCs significantly reduced their viability (Figure 9A). Given that ENPP2 was not expressed at all in TGF-β1-induced PDL fibroblasts, its absence could be considered equivalent to a complete knockdown of ENPP2 (Figure 4A, lane two and Figure 9B, lane two in a). If ENPP2 expression was indispensable for cell survival, TGF-β1-induced PDL fibroblasts might exhibit lethality. Surprisingly, PDL fibroblasts did not impact cell proliferation (Figure 9B, filled circles in b and Supplementary Figure S5). However, intriguingly, depletion of ENPP1 in PDL fibroblasts, where ENPP2 was absent, led to inhibited cell growth (Figure 9B, lane three in a and open rhomb in b). Conversely, when TGF-β1 signaling pathway was suppressed by SB431542, ENPP1 levels decreased while ENPP2 expression resurged to high level (Figure 9B, lane four in a), and cell growth proceeded normally (Figure 9B, filled circle in b and Supplementary Figure S4). Under these conditions, when ENPP2 was depleted by siRNA, meaning neither ENPP1 nor ENPP2 was expressed (Figure 9B, lane five in a), the cells exhibited defects (Figure 9B, open circle in b and Supplementary Figure S4).

The primary function of ENPP1 and ENPP2 enzymes were associated with pyrophosphate (PPi) and lysophosphatidic acid (LPA), respectively. With the addition of PPi and LPA in PDL fibroblasts depleted ENPP1, however, cell viability improved in both cases (Figure 9C, filled triangle and circle). Conversely, when ENPP2 expression was inhibited in cells blocked in TGF-β1 signaling pathway, the cells died (Figure 9C, open rhomb). When LPA was introduced, cell survival partially recovered (Figure 9C, open circles). However, PPi did not impact the lethality of ENPP2-depleted cells (Figure 9C, open triangles). These findings indicate that ENPP1 and ENPP2 complement each other in cell proliferation and viability maintenance. While ENPP2 negatively regulates TGF-β1-induced fibroblast differentiation, it is essential for cell survival in undifferentiated hPDLSCs and osteo/cementoblasts, where ENPP1 was absent, ENPP2 activity seems crucial for cell survival.

## Discussion

TGF-β1 is a representative factor that induces fibroblastic differentiation of PDLSCs. In our previous study, we validated that exposure to a low concentration of TGF-β1 effectively stimulated fibroblast differentiation, as evidenced by upregulation in PDL fibroblastic markers (Hyun et al., 2017; Lim et al., 2020) (Figure 1). Before conducting this investigation, we procured numerous monoclonal antibodies targeting cell surface molecules specific to PDL fibroblast through decoy immunization. The newly developed anti-PDL13 antibody identified ENPP1 as its antigen (Figure 2). ENPP1 was observed to accumulate on the surface of PDL fibroblasts upon differentiation induced by TGF-β1 treatment (Figure 3). The expression of ENPP1 protein significantly increased in PDL fibroblasts, and decreased upon simultaneous treated with SB431542, a TGF-β1 receptor inhibitor. These findings suggest the potential utility of ENPP1 as a surface marker for PDL fibroblasts.

A healthy periodontal ligament can prevent excessive bone formation and maintain hard-soft tissue homeostasis. It is crucial to avoid inappropriate calcification during stem cell mediated PDL regeneration. Numerous studies have demonstrated PPi inhibits calcification *in vitro.* Therefore, the upregulation of ENPP1, an enzyme responsible for PPi production, appears to be a critical event in PDL differentiation (Murshed et al., 2005; Addison et al., 2007). A deficiency in extracellular PPi promotes excessive hydroxyapatite formation; while increased PPi inhibits hydroxyapatite formation and mineralization (Foster et al., 2012). As depicted in Figure 5, treatment with PPi induced differentiation into PDL fibroblasts. Contrastingly, blocking the function of ENPP1 using siRNA and an anti-PDL13 antibody inhibited PDL fibroblast differentiation and increased mineralization (Figures 7, 8; Supplementary Figure S4). These findings underscore the crucial role of ENPP1 in TGF-β1-induced fibroblastic differentiation of hPDLSCs.

In contrast to ENPP1, TNAP hydrolyzes PPi to Pi, facilitating the formation of hydroxyapatite (Hessle et al., 2002; Zweifler et al., 2015). In this investigation, the extent of mineral formation was assessed by examining the correlation between ENPP1 and TNAP. As ENPP1 expression increased, TNAP expression and mineral formation decreased (Figures 3–5). This illustrates that ENPP1 and TNAP regulate differentiation by modulating mineralization through contrasting expression patterns. Thus, TNAP expression serves as an indicator to verify the inhibition of ENPP1 and the initiation of calcification.

ENPP2, a member of the same enzyme family as ENPP1, functions by hydrolyzing lysophosphatidylcholine (LPC) into lysophosphatidic acid (LPA), aside from its PPi-forming activity, which has a high KM value. It plays roles in various cellular processes, including proliferation, survival, and migration (Hama et al., 2004; Perrakis and Moolenaar, 2014). Apart from its recognized function in cancer cells, the involvement of ENPP2 in hard and soft tissue differentiation remains unclear. Through RNA-seq analysis, in our previous investigation, we identified that ENPP1 was one of the cell surface factors that increased in PDL fibroblasts, while ENPP2 expression was particularly elevated in osteo/cementoblasts (Mun et al., 2022). Thus, we hypothesized that ENPP2 and ENPP1 might exert contrasting effects on hPDLSC differentiation. Indeed, ENPP2 expression increased upon BMP7 treatment, but was completely absent during fibroblastic differentiation induced by TGF-β1 (Figure 5A). Moreover, ENPP2 overexpression hindered PDL fibroblast differentiation (Figures 6A, B). This result indicated the ENPP2’s contrasting role compared to ENPP1. By the suppression of the differentiation of hPDLSCs into PDL fibroblasts, ENPP2 was observed to promote osteo/cementoblastic differentiation. However, even with a significant increase in ENPP2 expression, calcification was not promoted in the absence of TNAP (Figure 6C, 3 and 4).

Interestingly, both ENPP1 and ENPP2 influence the survival of undifferentiated and differentiated hPDLSCs. When ENPP2 expression was suppressed in undifferentiated cells, cell proliferation dramatically decreased (Figures 9A). However, inhibiting ENPP1 expression in undifferentiated cells did not lead to decreased cell proliferation (data not shown). Given that ENPP2 is not expressed when ENPP1 is upregulated during fibroblastic differentiation, and conversely, ENPP1 is not expressed during osteo/cementoblastic differentiation when ENPP2 is expressed, the introduction of siRNA targeting each gene allows for the knockdown of both ENPP1 and ENPP2 in respective conditions. Remarkably, upon suppressing the expression of both ENPP1 and ENPP2, a significant decrease in cell proliferation was observed (Figure 9B, open shapes in b). These results underscore the significance of the enzymatic activities of ENPP1 and ENPP2 for cell survival, demonstrating that survival is feasible even with only one of them. Consequently, they suggest the potential for complementarity between the two enzymes.

Numerous previous studies have highlighted the significance of extracellular LPA concentration, that is primarily produced by ENPP2, in cell survival and proliferation. Despite the low efficiency, ENPP1 produced not only PPi but also LPA in our *in vitro* enzymatic assay (Supplementary Figure S6). In undifferentiated hPDLSCs and osteo/cementoblasts, where ENPP1 is absent, ENPP2 activity emerges as crucial for cell survival.

**FIGURE 10:**
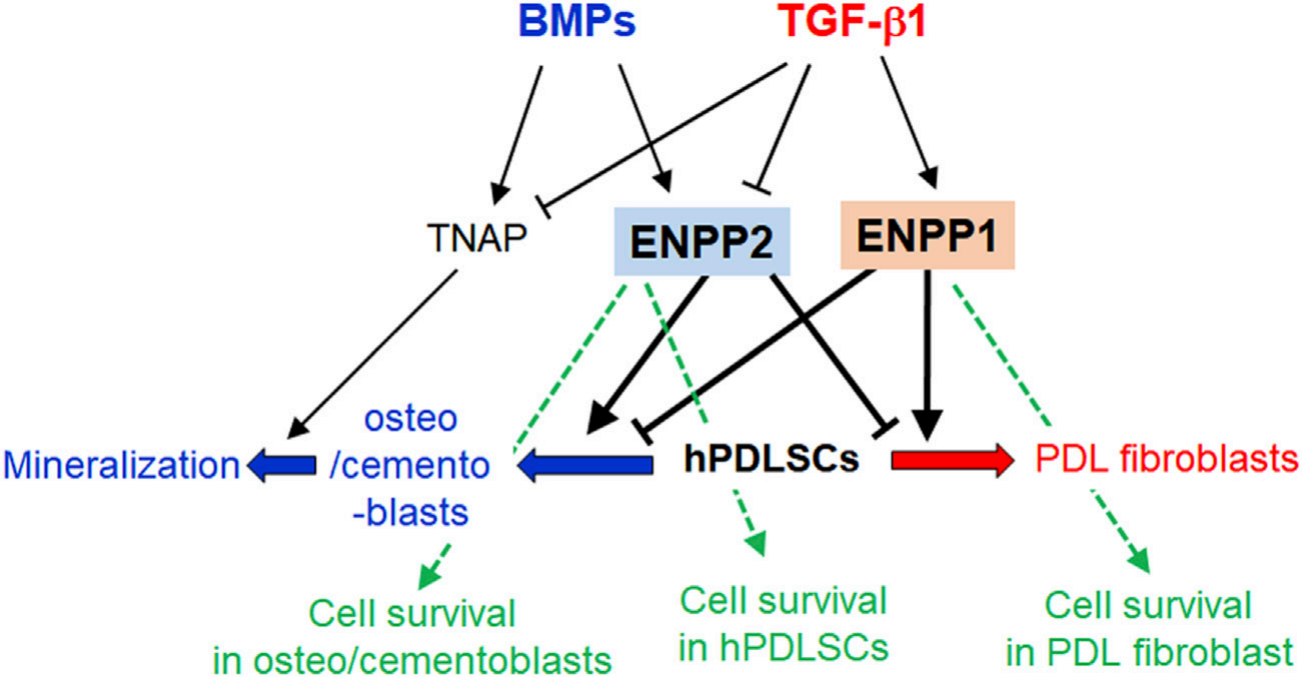
Schematic illustration of the roles of ENPP1, ENPP2, and TNAP in differentiations and mineralization of hPDLSCs. In TGF-β1-induced PDL fibroblastic differentiation, ENPP1 expression increases but TNAP and ENPP2 completely decrease. Conversely, ENPP1 is not expressed in BMP7-mediated osteo/cementoblastic differentiation, but TNAP is increased. TNAP expression is essential for induction of mineralization. For maintaining PDL fibroblastic differentiation, ENPP2 expression is blocked as well as TNAP. ENPP1 and ENPP2 act in opposition to each other during the differentiation process of hPDLSCs. In osteo/cementoblasts and undifferentiated hPDLSCs, ENPP2 is important for cell survival. On the other hand, ENPP1 is involved in cell survival during PDL fibroblast differentiation induced by TGF-β1, in which ENPP2 is completely inhibited.

The schematic diagram in Figure 10 provides a concise summary of the roles played by ENPP1 and ENPP2 in the cell proliferation, differentiation into hard and soft tissue, and mineralization of hPDLSCs. Further clarification of the antagonistic roles of these two proteins in hPDLSCs differentiation, along with their molecular mechanisms, will be explored through additional experiments.

## Data Availability

The original contributions presented in the study are included in the article/Supplementary Material, further inquiries can be directed to the corresponding author.
