# Supplementary figures and images for: Regulation of TGF-β1-induced fibroblast differentiation of human periodontal ligament stem cells through the mutually antagonistic action of ectonucleotide pyrophosphatase/phosphodiesterase 1 and 2
Source: Front Cell Dev Biol. 2024 Sep 3;12:1426762. doi: 10.3389/fcell.2024.1426762 (PMC11405333; doi:10.3389/fcell.2024.1426762)

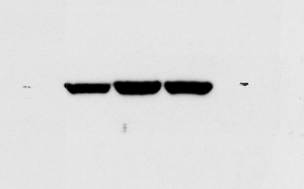

Supplement: Supplementary file 1 [file DataSheet3.ZIP › 7A-3.tif]

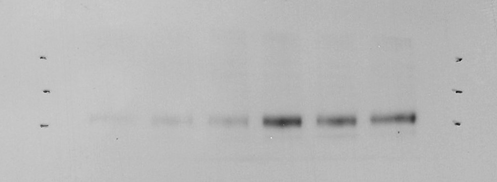

Supplement: Supplementary file 1 [file DataSheet3.ZIP › 8A-1.tif]

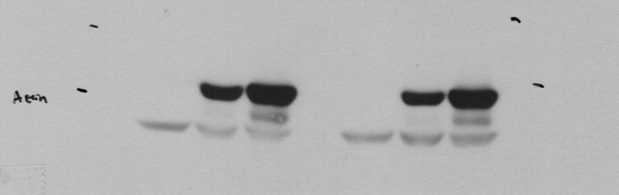

Supplement: Supplementary file 1 [file DataSheet3.ZIP › 8A-2.tif]

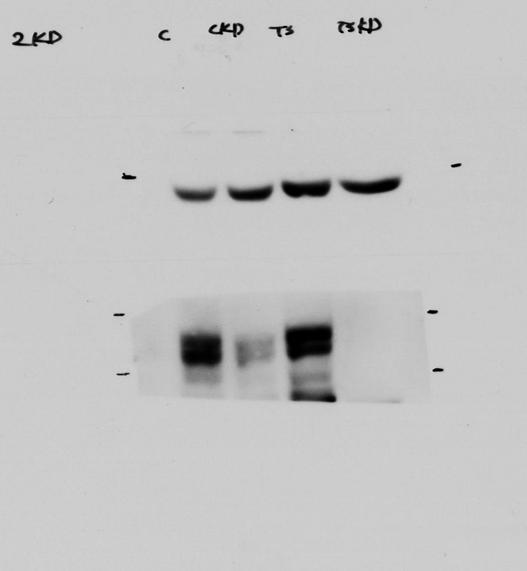

Supplement: Supplementary file 1 [file DataSheet3.ZIP › 9Aa.tif]

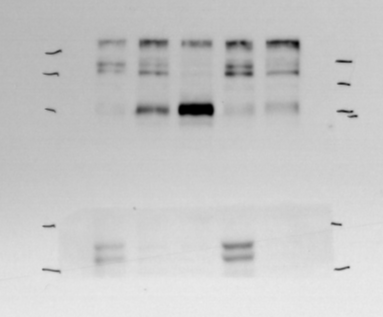

Supplement: Supplementary file 1 [file DataSheet3.ZIP › 9Ba-1&2.tif]

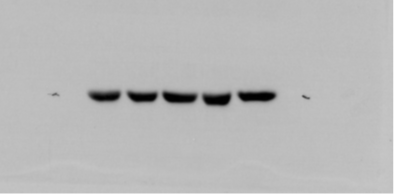

Supplement: Supplementary file 1 [file DataSheet3.ZIP › 9Ba-3.tif]

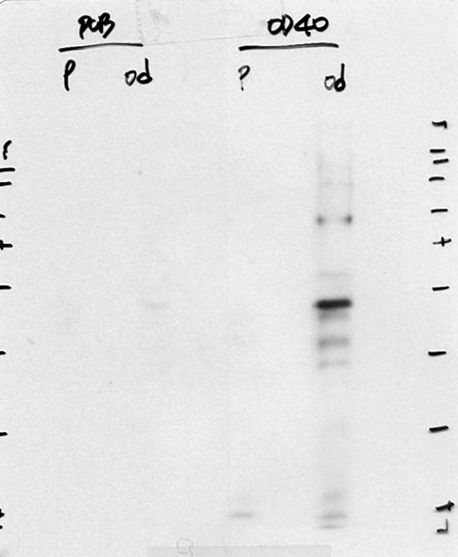

Supplement: Supplementary file 1 [file DataSheet3.ZIP › 2A.tif]

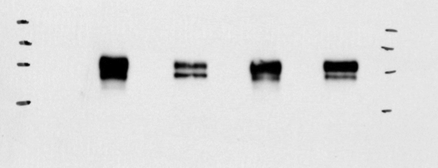

Supplement: Supplementary file 1 [file DataSheet3.ZIP › 2Ba-1.tif]

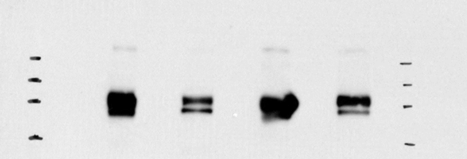

Supplement: Supplementary file 1 [file DataSheet3.ZIP › 2Ba-2.tif]

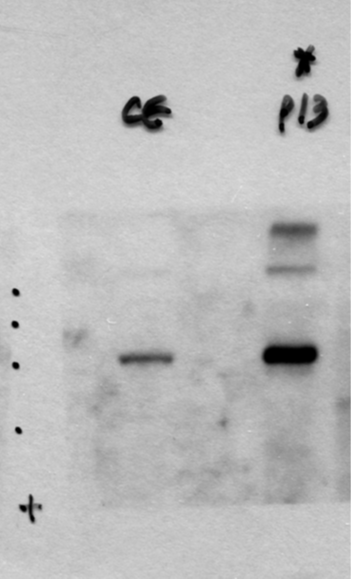

Supplement: Supplementary file 1 [file DataSheet3.ZIP › 2Bb.tif]

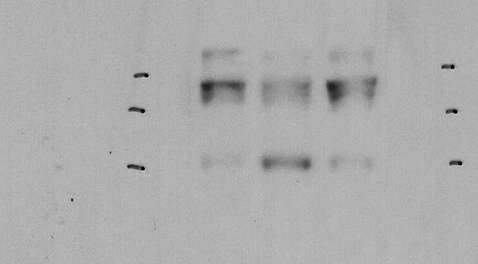

Supplement: Supplementary file 1 [file DataSheet3.ZIP › 3A-1.tif]

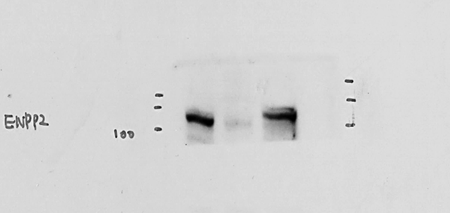

Supplement: Supplementary file 1 [file DataSheet3.ZIP › 3A-2.tif]

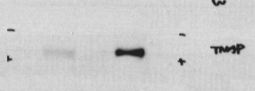

Supplement: Supplementary file 1 [file DataSheet3.ZIP › 3A-3.tif]

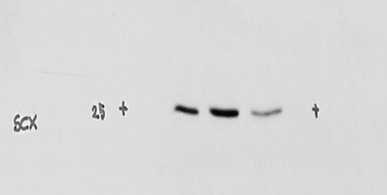

Supplement: Supplementary file 1 [file DataSheet3.ZIP › 3A-4.tif]

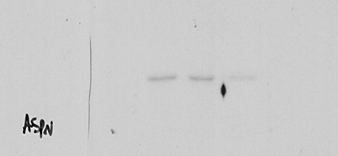

Supplement: Supplementary file 1 [file DataSheet3.ZIP › 3A-5.tif]

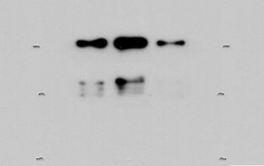

Supplement: Supplementary file 1 [file DataSheet3.ZIP › 3A-6.tif]

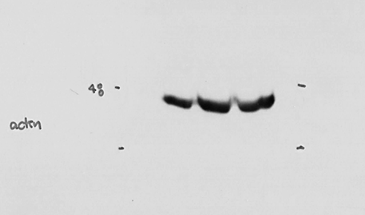

Supplement: Supplementary file 1 [file DataSheet3.ZIP › 3A-7.tif]

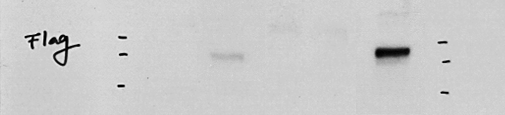

Supplement: Supplementary file 1 [file DataSheet3.ZIP › 4A-1.tif]

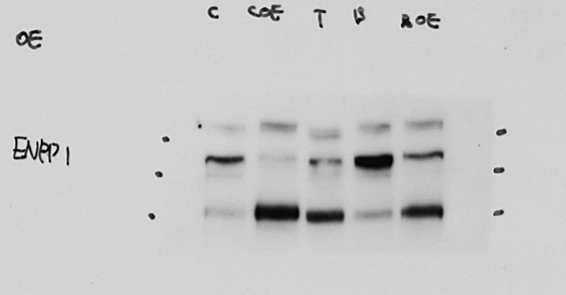

Supplement: Supplementary file 1 [file DataSheet3.ZIP › 4A-2.tif]

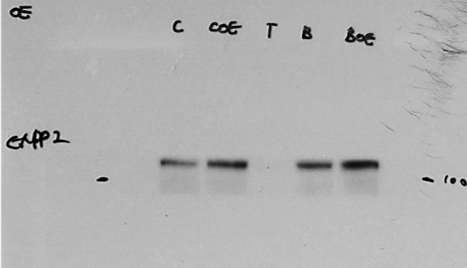

Supplement: Supplementary file 1 [file DataSheet3.ZIP › 4A-3.tif]

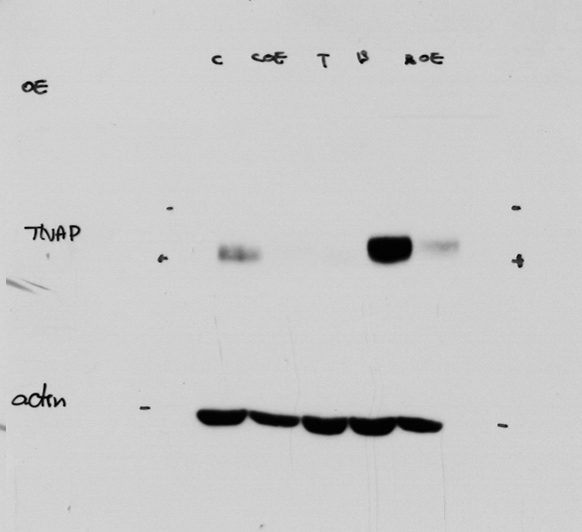

Supplement: Supplementary file 1 [file DataSheet3.ZIP › 4A-4&5.tif]

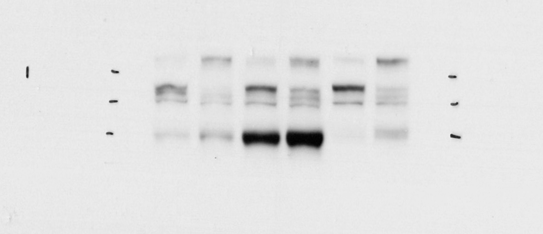

Supplement: Supplementary file 1 [file DataSheet3.ZIP › 5A-1.tif]

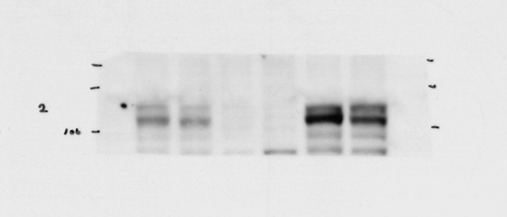

Supplement: Supplementary file 1 [file DataSheet3.ZIP › 5A-2.tif]

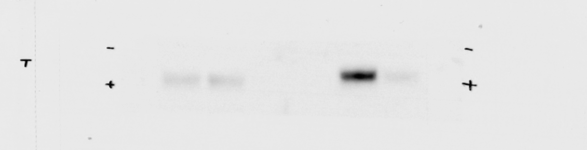

Supplement: Supplementary file 1 [file DataSheet3.ZIP › 5A-3.tif]

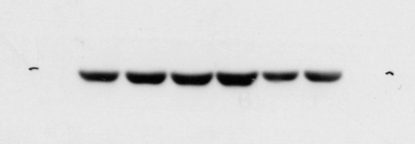

Supplement: Supplementary file 1 [file DataSheet3.ZIP › 5A-4.tif]

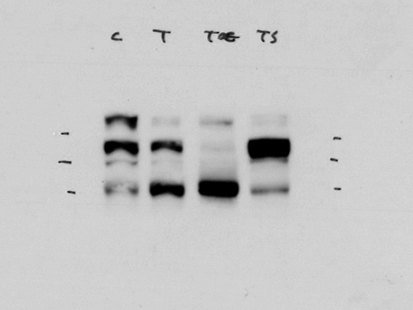

Supplement: Supplementary file 1 [file DataSheet3.ZIP › 6A-1.tif]

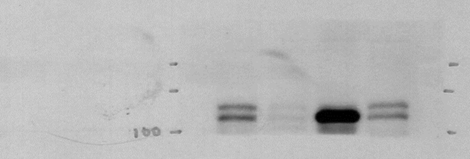

Supplement: Supplementary file 1 [file DataSheet3.ZIP › 6A-2.tif]

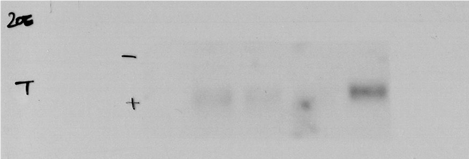

Supplement: Supplementary file 1 [file DataSheet3.ZIP › 6A-3.tif]

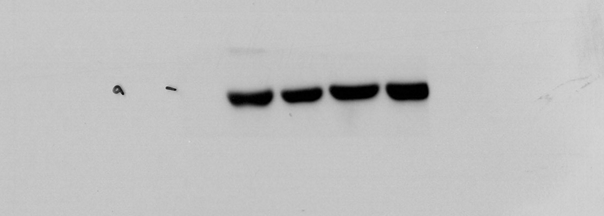

Supplement: Supplementary file 1 [file DataSheet3.ZIP › 6A-4.tif]

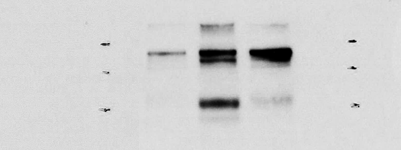

Supplement: Supplementary file 1 [file DataSheet3.ZIP › 7A-1.tif]

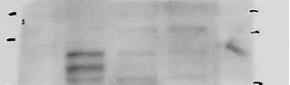

Supplement: Supplementary file 1 [file DataSheet3.ZIP › 7A-2.tif]

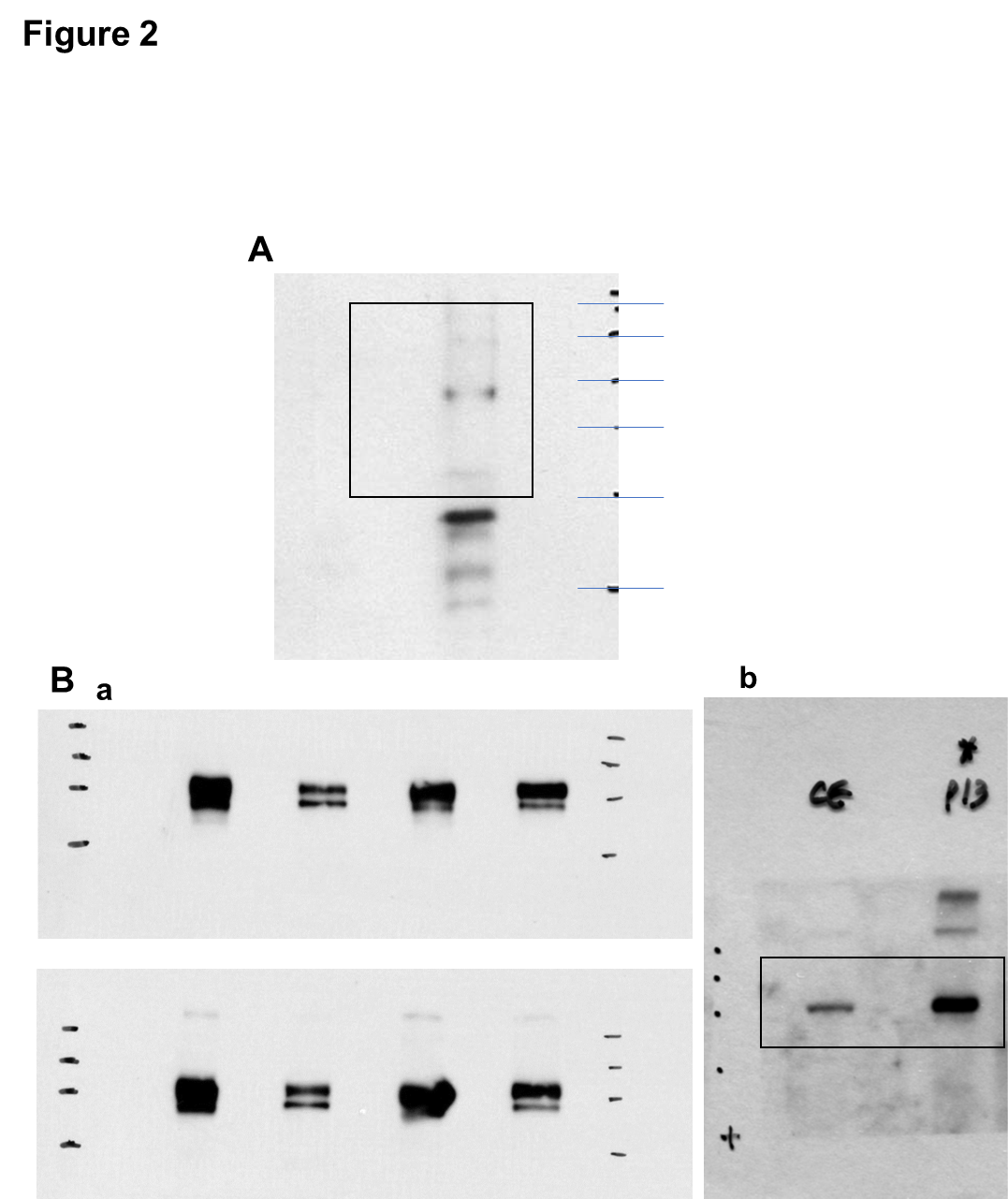


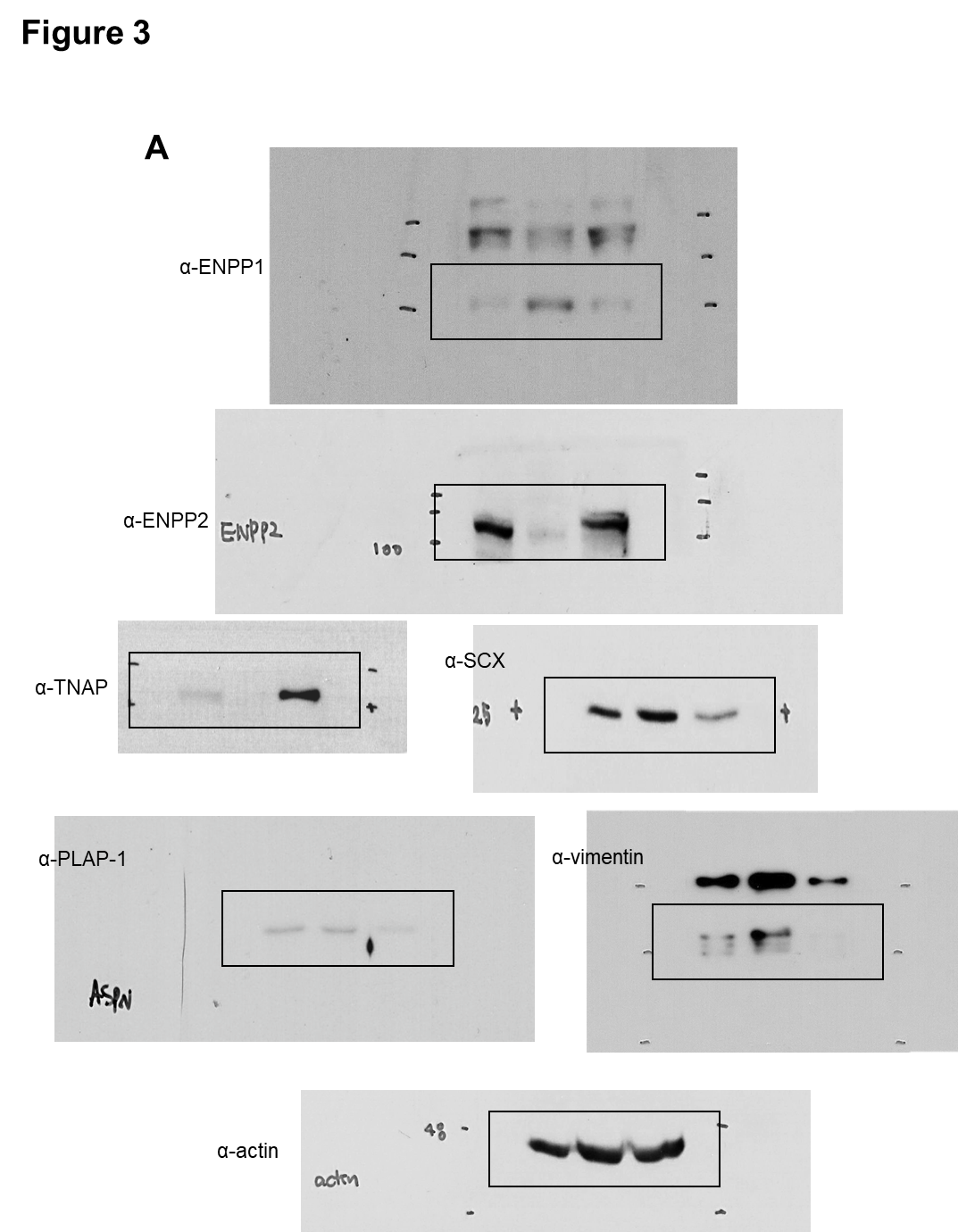


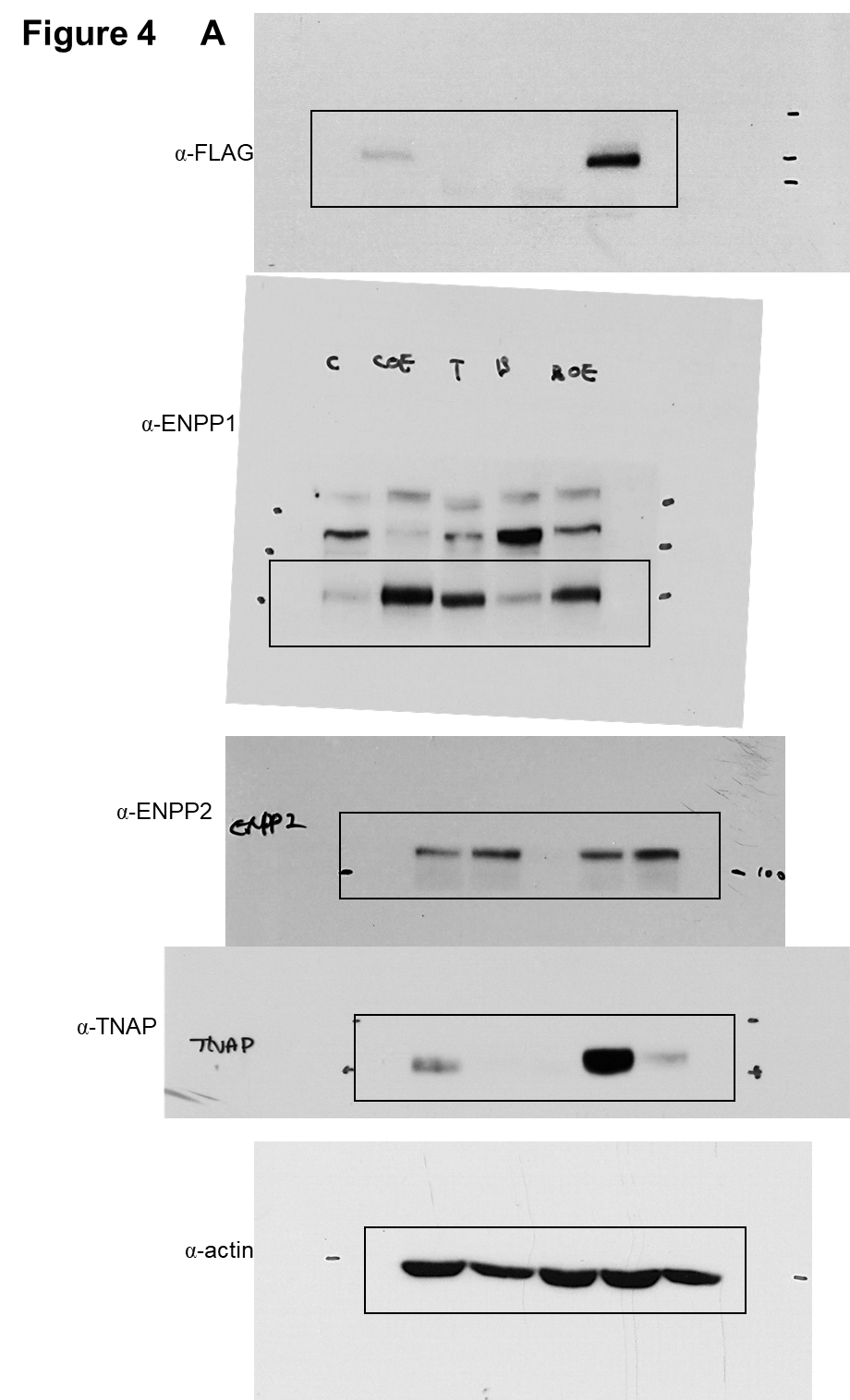


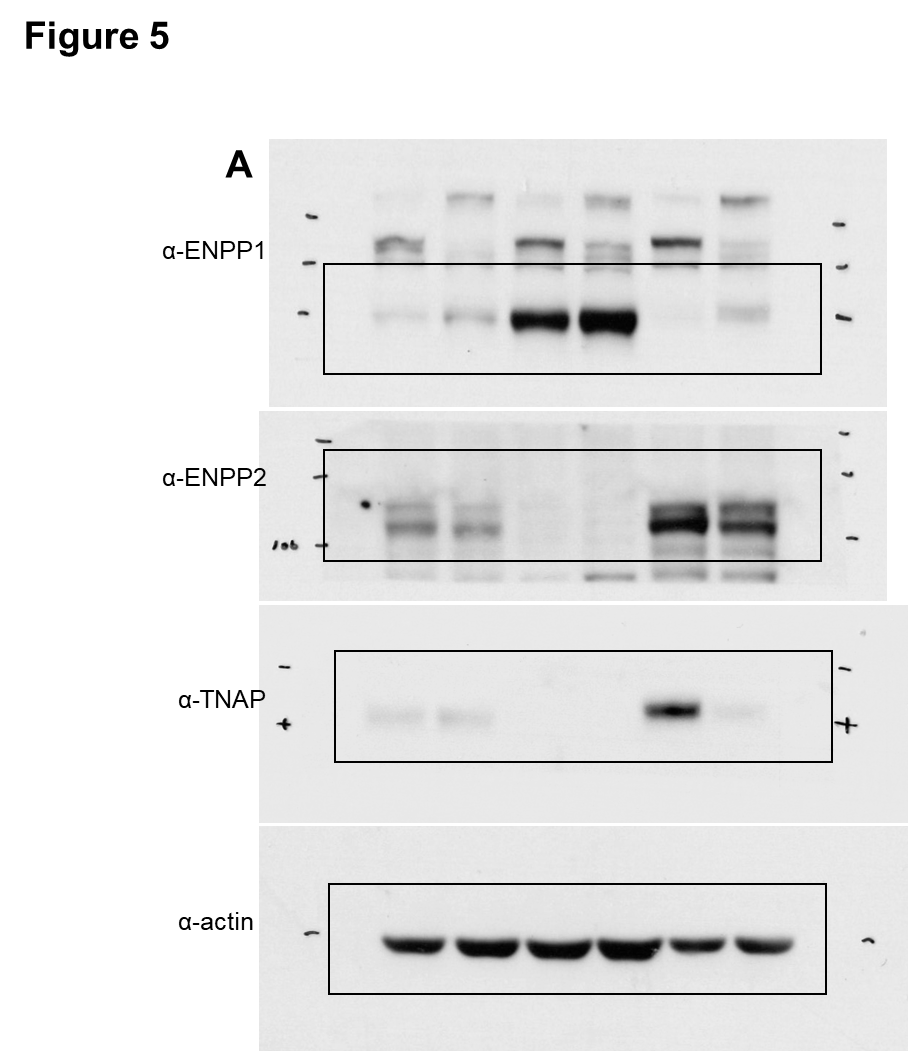


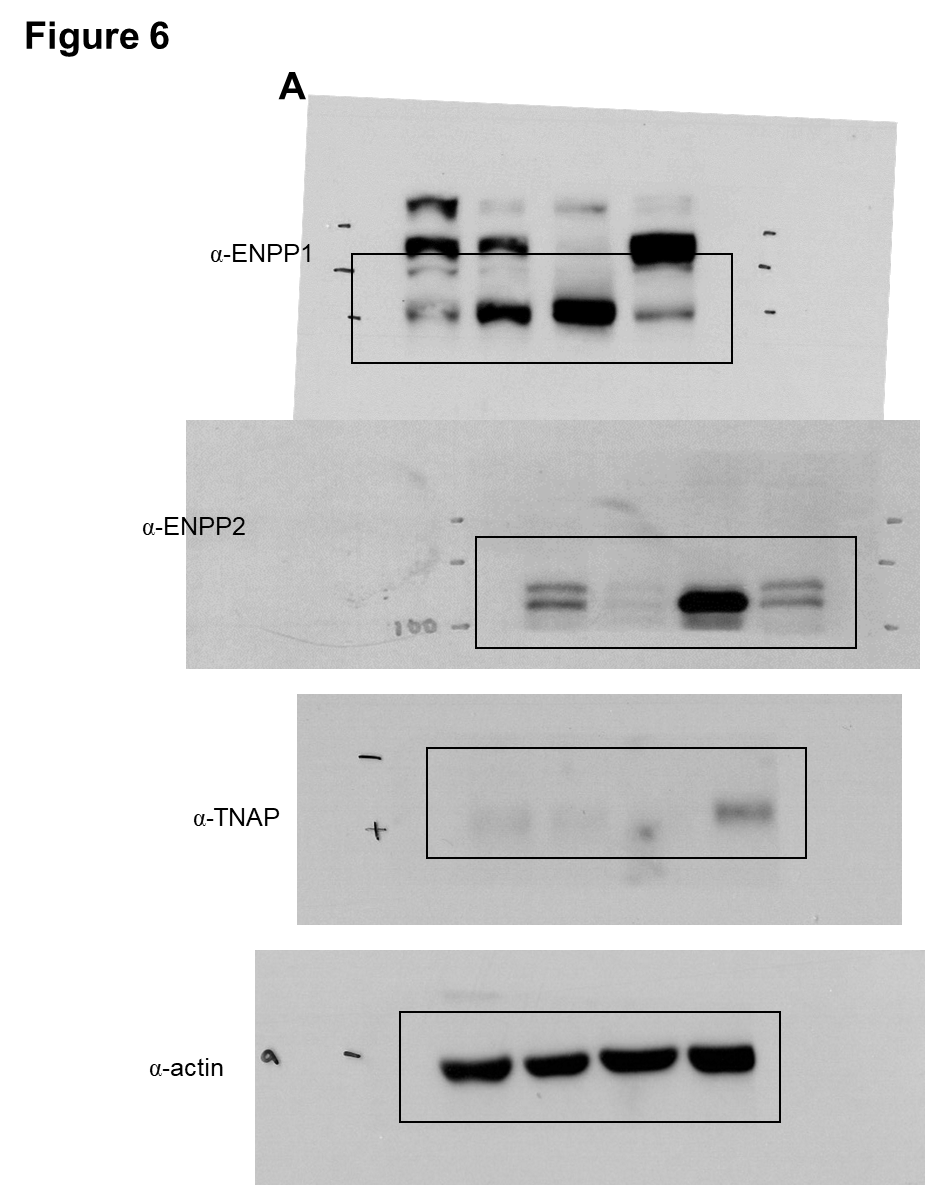


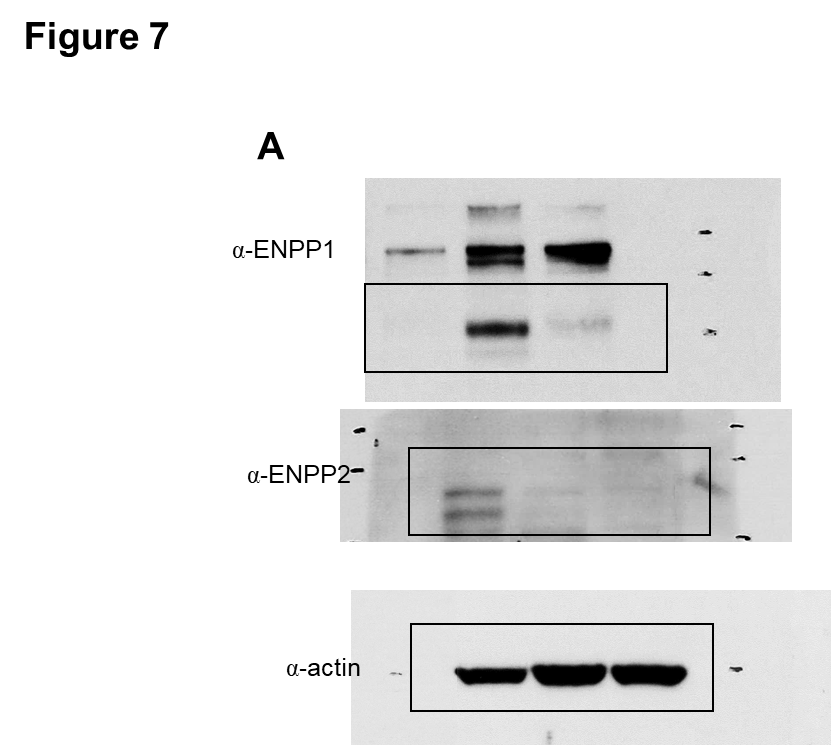


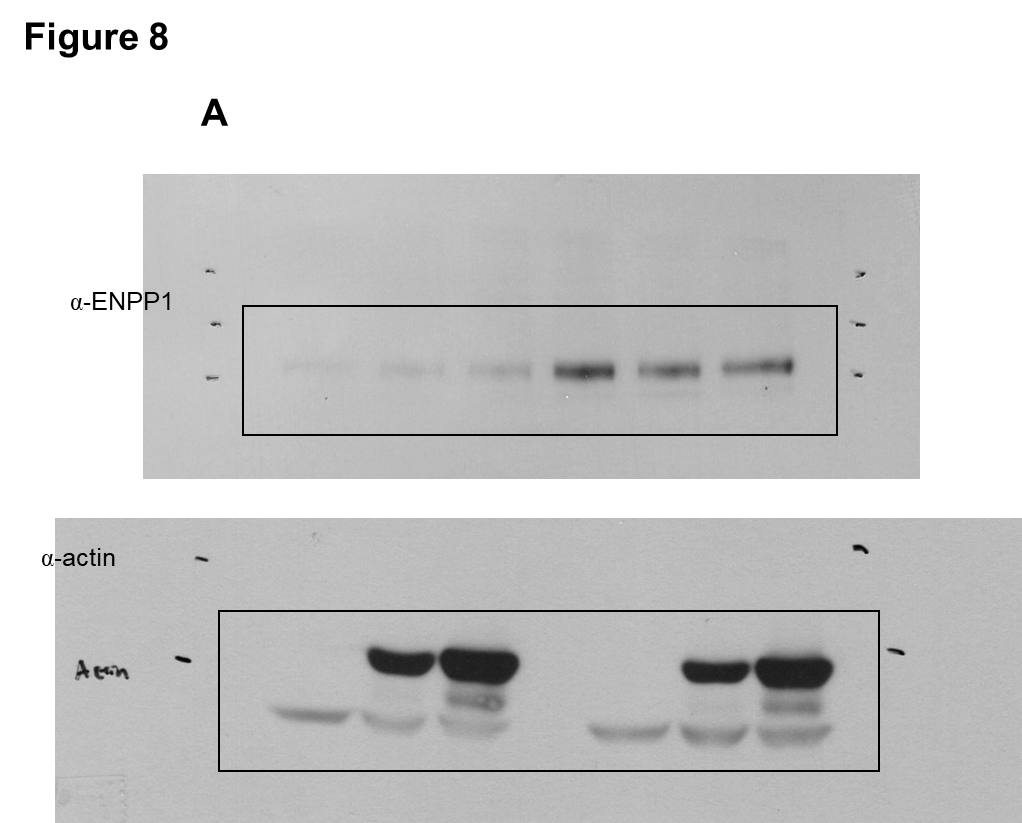


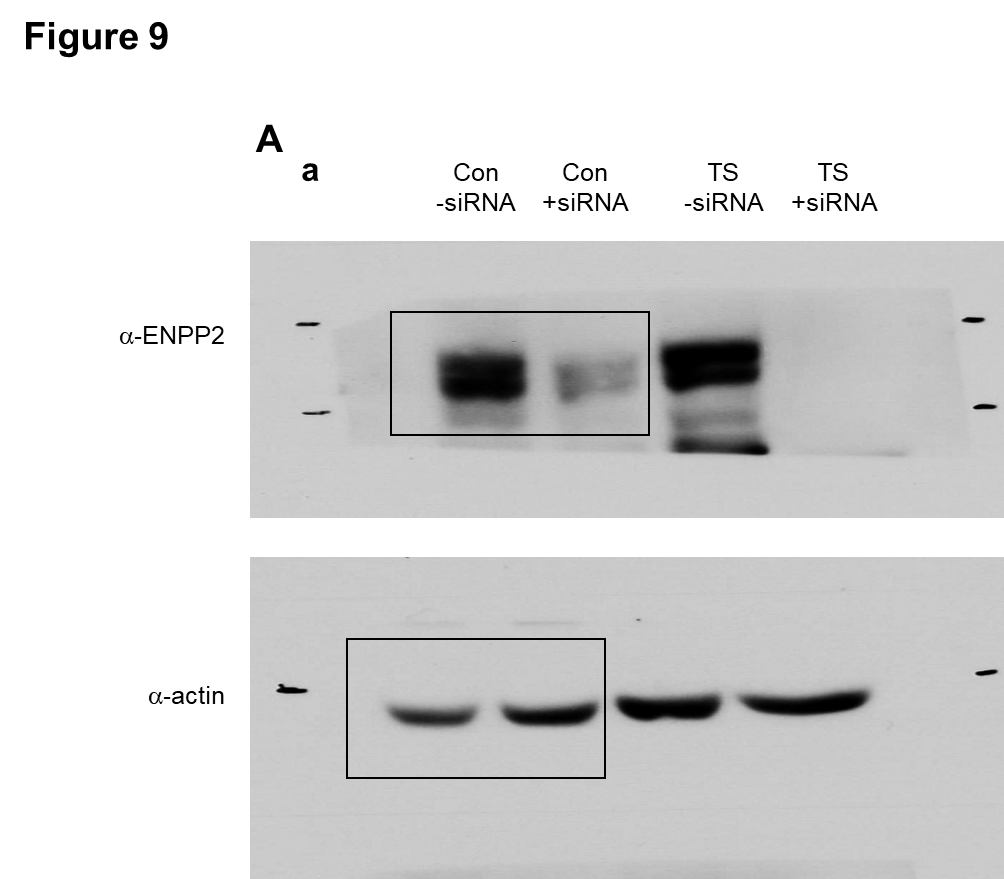


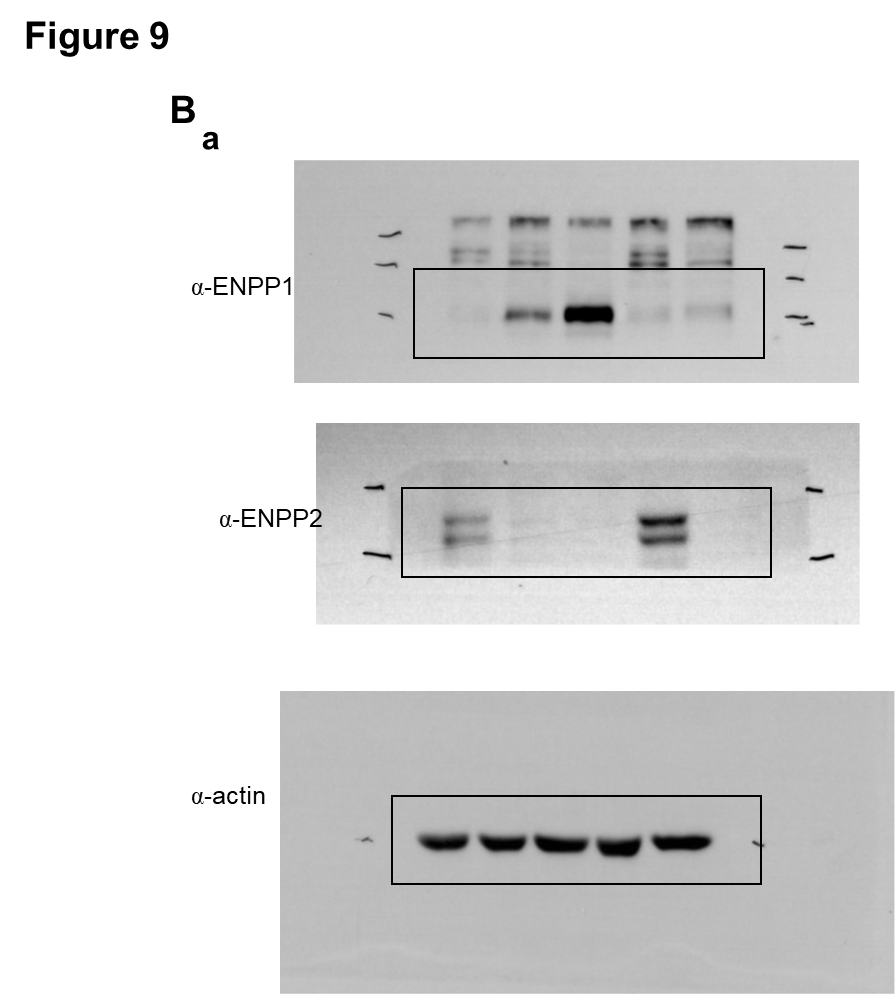

Supplement: Supplementary file 3 [file DataSheet2.DOCX]
