# Supplementary material for: Regulation of TGF-β1-induced fibroblast differentiation of human periodontal ligament stem cells through the mutually antagonistic action of ectonucleotide pyrophosphatase/phosphodiesterase 1 and 2
Source: Front Cell Dev Biol. 2024 Sep 3;12:1426762. doi: 10.3389/fcell.2024.1426762 (PMC11405333; doi:10.3389/fcell.2024.1426762)
Supplement: Supplementary file 2 [file Table1.DOCX]

Supplementary Table 1. Primers used for the quantitative real-time-PCR (qPCR).

| Gene |  | Primer sequence |
| --- | --- | --- |
| GAPDH | Forward  Reverse | 5’-GTATGACAACAGCCTCAAGAT-3’  5’-CCTTCCACGATACCAAAGTT-3’ |
| Cementum protein 1 (CEMP1) | Forward  Reverse | 5’- GATCAGCATCCTGCTCATGTT-3’  5’-AGCCAAATGACCCTTCCATTC-3’ |
| Osterix (OSX) | Forward  Reverse | 5′-GAAGGGAGTGGTGGAGCCAAAC-3'  5′-ATTAGGGCAGTCGCAGGAGGAG-3' |
| Scleraxis (SCX) | Forward  Reverse | 5’-AGAAAGTTGAGCAAGGACC-3’  5’-CTGTCTGTACGTCCGTCT-3’ |
| Periodontal ligament-associated protein-1  (PLAP-1) | Forward  Reverse | 5’-TTGACCTCAGTCCCAACCAA-3’  5’-TCGTTAGCTTGTTGTTGTTCAG-3’ |
