# Supplementary material for: Regulation of TGF-β1-induced fibroblast differentiation of human periodontal ligament stem cells through the mutually antagonistic action of ectonucleotide pyrophosphatase/phosphodiesterase 1 and 2
Source: Front Cell Dev Biol. 2024 Sep 3;12:1426762. doi: 10.3389/fcell.2024.1426762 (PMC11405333; doi:10.3389/fcell.2024.1426762)
Supplement: Supplementary file 4 [file DataSheet1.DOCX]

**Supplementary Figure 1**

**
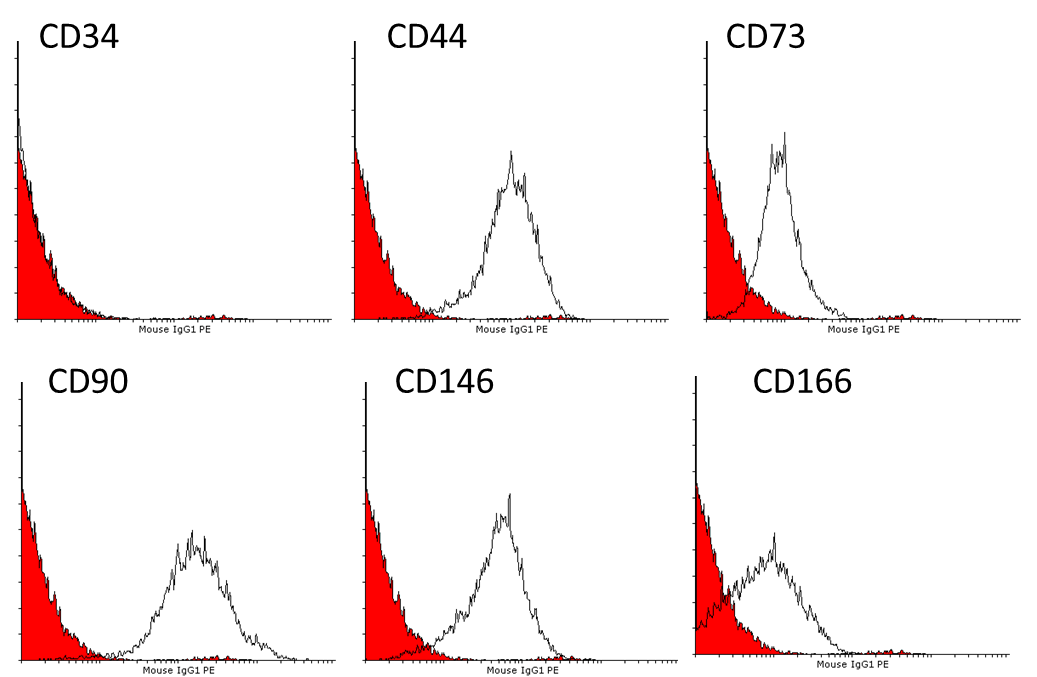
**

**Supplementary Figure 1.** Immunophenotyping of human periodontal ligament stem cells. Cells were harvested using trypsin-free dissociation buffer (Millipore) and suspended in PBS containing 5 % fetal bovine serum at 1 x 10^6^ cells/ml of concentration. For immunophenotyping, antibodies against the following representative mesenchymal stem cell surface antigens were treated with cells: CD44, CD73, CD90, CD146, and CD166. As a negative marker, anti-CD34 antibody was used. Anti-human IgG was used as negative control (filled curves), and the fluorescent intensity of the specific antibody binding was indicated by open curves.

**Supplementary Figure 2**


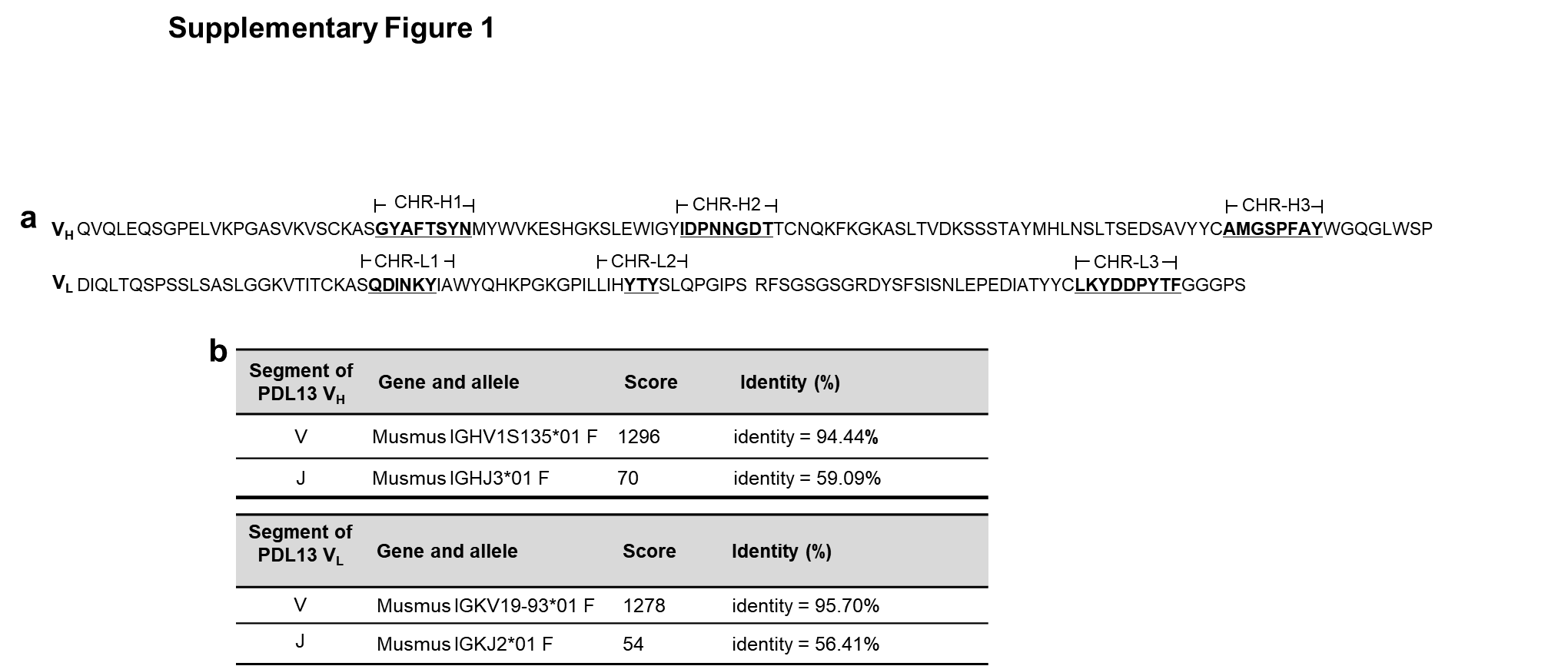


**Supplementary Figure 2.** Sequence information and identity analysis of the variable regions of anti-PDL13 antibody. **a,** Amino acid sequences of mouse immunoglobulin heavy and light chain variable regions. Three complementarity determining regions in heavy and light chains (CDR-H1~3 and CDR-L1~3) were shown in bold underlined. **b,** IMGT/V-QUEST database-based antibody similarity analysis.

**Supplementary Figure 3**

**
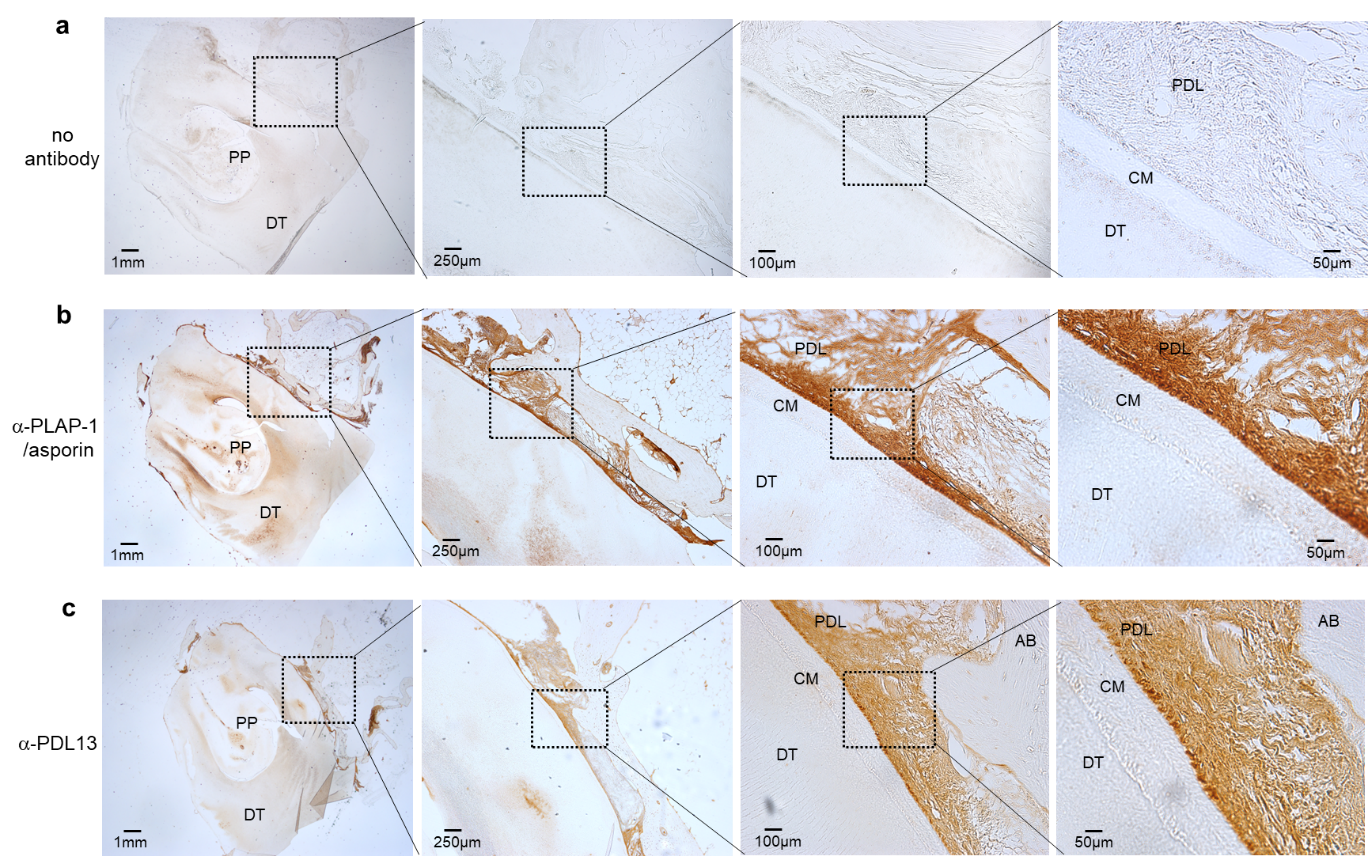
**

**Supplementary Figure 3.** Immunohistochemistry using tooth slice. Each slice of tooth root was stained with the indicated antibody. In panels, PP, DT, CM, PDL, and AB indicated pulp, dentin, cementum, periodontal ligament, and alveolar bone, respectively.

**Supplementary Figure 4**


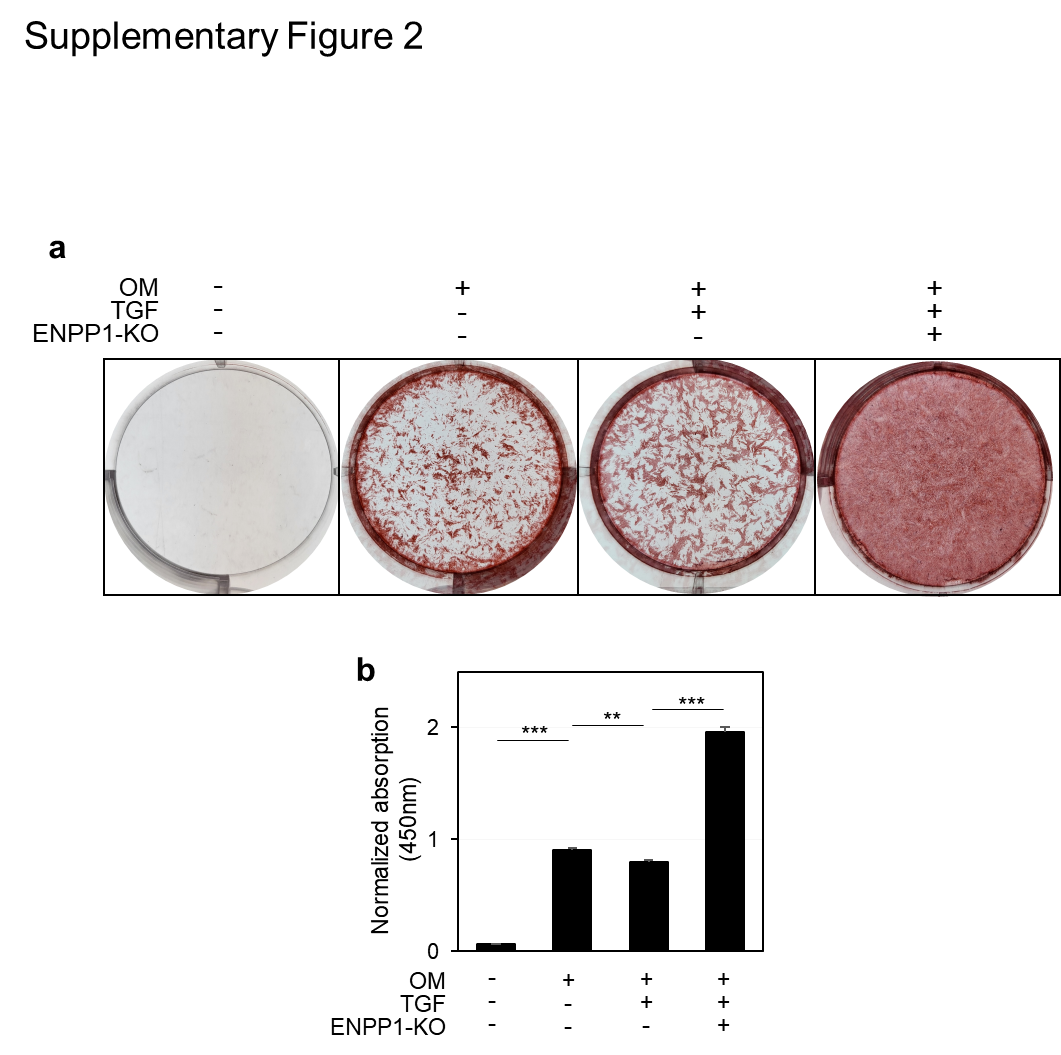


**Supplementary Figure 4.** Mineralization was increased by ENPP1 depletion. Panels indicated the calcified mineral amounts in undifferentiated hPDLSCs in normal media, undifferentiated hPDLSCs in mineral induction media (OM), TGF-β1-induced PDL fibroblasts, and fibroblasts with siRNA (ENPP1-KO) in that order. Mineralization was analyzed by alizarin staining as described in Materials and Methods. a, dyed photo of alizarin red staining; b, colorimetric detection at 405 nm of extracts of calcified mineral at low pH.

**Supplementary Figure 5**

**
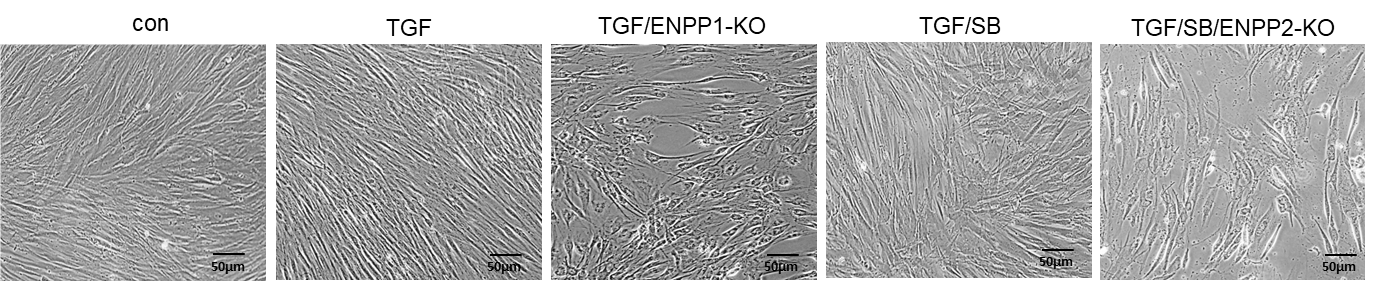
**

**Supplementary Figure 5.** Cell viability of cells depleted of ENPP1 in the absence of ENPP2 and vice versa. Photos indicated to undifferentiated hPDLCs, TGF-β1-induced PDL fibroblasts, PDL fibroblasts with depleted ENPP1, PDL fibroblasts treated with SB431542, and PDL fibroblasts treated with SB431542 and ENPP2 siRNA in that order.

**Supplementary Figure 6**


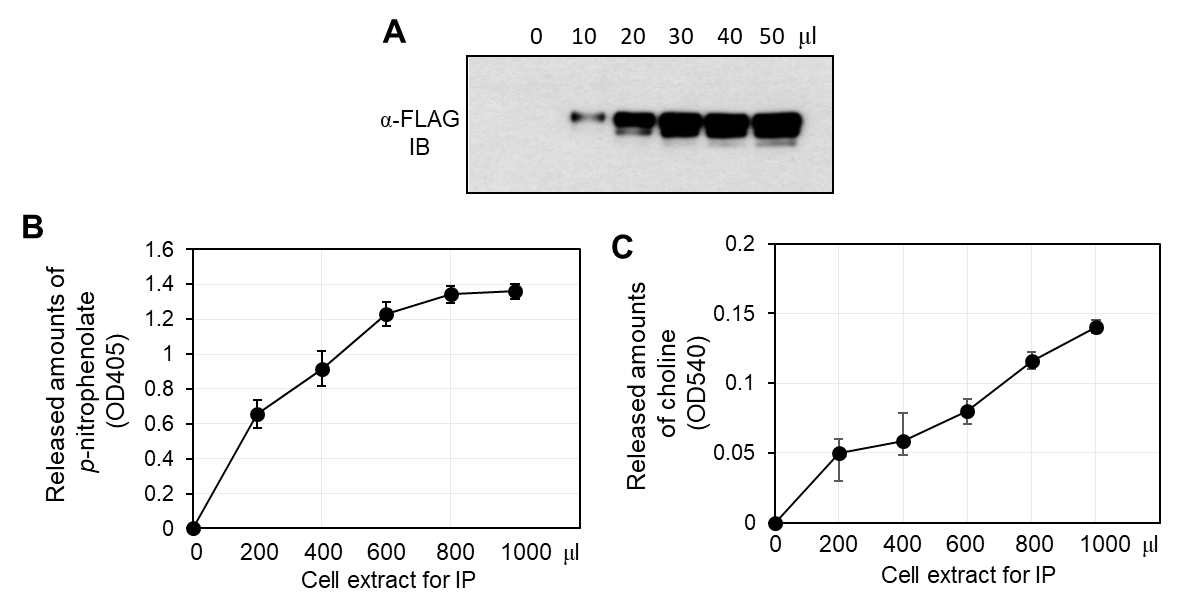


**Supplementary Figure 6.** *In vitro* enzyme activity of ENPP1. (A) The amounts of ENPP1 ectopically expressed in HeLa cells. Ectopic ENPP1 levels in cell extract were confirmed in the indicated volumes (0-50 μl) using anti-FLAG antibody. (B) Nucleotide phosphodiesterase assay. The indicated amounts of cell extracts (1-1000 μl in x-axis) were used for ENPP1 immunoprecipitation. Immunoprecipitates (IP) were applied for the enzyme assay of nucleotide phosphodiesterase following the methods in previous reports (Cimpean et al., 2004; Jansen et al., 2009). The amount of released p-nitrophenolate was estimated by optical density at 405 nm. (C) Lysophospholipase D assay. The indicated amounts of cell extracts (1-1000 μl in x-axis) were used for ENPP1 immunoprecipitation. Immunoprecipitates (IP) were applied for the enzyme assay of Lysophospholipase following the methods in previous reports (Cimpean et al., 2004; Jansen et al., 2009). The amount of released choline was estimated by optical density at 540 nm.

- Cimpean, A., et al. (2004) Biochem J. 231, 71-77
- Jansen, S., et al. (2009) J Biol Chem. 284, 14296-14302
